# The Impact of Visualization on Stroke Rehabilitation in Adults: A Systematic Review of Randomized Controlled Trials on Guided and Motor Imagery

**DOI:** 10.3390/biomedicines13030599

**Published:** 2025-03-01

**Authors:** Andrea Calderone, Alfredo Manuli, Francesca Antonia Arcadi, Annalisa Militi, Simona Cammaroto, Maria Grazia Maggio, Serena Pizzocaro, Angelo Quartarone, Alessandro Marco De Nunzio, Rocco Salvatore Calabrò

**Affiliations:** 1 Department of Clinical and Experimental Medicine, University of Messina, Piazza Pugliatti, 98122 Messina, Italy; 2 Physical Medicine and Rehabilitation Unit, AOU Policlinico Universitario in Messina, 98125 Messina, Italy; manulialfredo@gmail.com; 3 IRCCS Centro Neurolesi Bonino-Pulejo, S.S. 113 Via Palermo, C.da Casazza, 98124 Messina, Italy; francesca.arcadi@irccsme.it (F.A.A.); annalisa.militi@irccsme.it (A.M.); simona.cammaroto@irccsme.it (S.C.); mariagrazia.maggio@irccsme.it (M.G.M.); angelo.quartarone@irccsme.it (A.Q.); roccos.calabro@irccsme.it (R.S.C.); 4 Laboratory of Bioengineering, Department of Electrical, Computer and Biomedical Engineering, University of Pavia, 27100 Pavia, Italy; 5 Department of Health, LUNEX University of Applied Sciences, 50, Avenue du Parc des Sports, 4671 Differdange, Luxembourg; adenunzio@lunex.lu; 6 Luxembourg Health & Sport Sciences Research Institute A.s.b.l., 50, Avenue du Parc des Sports, 4671 Differdange, Luxembourg

**Keywords:** guided imagery, motor imagery, stroke rehabilitation, motor recovery, neurorehabilitation, post-stroke

## Abstract

**Background/Objectives:** Guided imagery techniques, which include mentally picturing motions or activities to help motor recovery, are an important part of neuroplasticity-based motor therapy in stroke patients. Motor imagery (MI) is a kind of guided imagery in neurorehabilitation that focuses on mentally rehearsing certain motor actions in order to improve performance. This systematic review aims to evaluate the current evidence on guided imagery techniques and identify their therapeutic potential in stroke motor rehabilitation. **Methods:** Randomized controlled trials (RCTs) published in the English language were identified from an online search of PubMed, Web of Science, Embase, EBSCOhost, and Scopus databases without a specific search time frame. The inclusion criteria take into account guided imagery interventions and evaluate their impact on motor recovery through validated clinical, neurophysiological, or functional assessments. This review has been registered on Open OSF with the following number: DOI 10.17605/OSF.IO/3D7MF. **Results:** This review synthesized 41 RCTs on MI in stroke rehabilitation, with 996 participants in the intervention group and 757 in the control group (average age 50–70, 35% female). MI showed advantages for gait, balance, and upper limb function; however, the RoB 2 evaluation revealed ‘some concerns’ related to allocation concealment, blinding, and selective reporting issues. Integrating MI with gait training or action observation (AO) seems to improve motor recovery, especially in balance and walking. Technological methods like brain–computer interfaces (BCIs) and hybrid models that combine MI with circuit training hold potential for enhancing functional mobility and motor results. **Conclusions:** Guided imagery shows promise as a beneficial adjunct in stroke rehabilitation, with the potential to improve motor recovery across several domains such as gait, upper limb function, and balance.

## 1. Introduction

In 1970, the World Health Organization (WHO) defined a stroke as ‘rapidly developed clinical signs of focal (or global) disturbance of cerebral function, lasting more than 24 h or leading to death, with no apparent cause other than of vascular origin’ [1,2]. According to the WHO, each year 12.2 million new cases are diagnosed, and 6.5 million deaths are reported around the globe [3,4]. Motor rehabilitation is essential for recovery after a stroke because neurological damage frequently results in major deficits in motor abilities, coordination, and movement [5,6,7]. Stroke survivors often face hemiparesis, muscle weakness, spasticity, and diminished fine motor skills, which can greatly impair their capacity to carry out daily tasks autonomously [8,9,10]. These motor impairments not only hinder physical capability but also lead to a deterioration in overall life quality, elevating the likelihood of depression, social seclusion, and extended disability [11,12]. Successful motor rehabilitation approaches seek to reinstate movement patterns, improve neuromuscular control, and foster neuroplasticity, which is the capacity of the nervous system to compensate for injury by strengthening existing connections, forming new synapses, and recruiting alternative neural networks to regain motor, sensory, or cognitive abilities. In recovery from stroke, neuroplasticity is one of the main mechanisms that underlie functional recovery by facilitating cortical reorganization and the recovery of lost motor function via experience-dependent synaptic modifications. The brain also undergoes a number of plastic alterations after a stroke, including dendritic sprouting, axonal remodeling, and cortical excitability changes, particularly in motor-related areas including the primary motor cortex (M1), premotor cortex, and supplementary motor area [13,14,15]. These changes are directed by therapeutic interventions to augment the brain’s inherent capacity to reorganize after an injury. Guided imagery methods, which include mentally imagining motions or activities to aid motor recovery, are an important component of neuroplasticity-based motor rehabilitation [16,17]. MI activates neural circuits similar to those engaged during actual movement execution, involving M1, the premotor cortex, the cerebellum, and the basal ganglia. This common neural representation indicates that MI may strengthen current motor pathways, elevate corticospinal excitability, and improve functional connectivity among motor and sensory areas [18]. Another significant neuroplastic mechanism associated with guided imagery is its capacity to improve sensory–motor integration. Because MI necessitates that individuals visualize and “experience” movement in their minds, it activates proprioceptive and kinesthetic processing routes, enhancing the connection between motor intentions and sensory input [19]. This integration is crucial for enhancing motor programs and maximizing motor control, especially in individuals rehabilitating from stroke, where sensorimotor impairments can pose a major obstacle to functional progress. These strategies are based on the idea that imagining a movement activates brain pathways similar to those used during actual physical performance, strengthening motor circuits even when no observable movement happens [20,21]. Guided imagery can be presented in several ways. A therapist, for example, might vocally lead a patient through an organized mental exercise, explaining each movement in detail and urging them to picture sensations like muscle contraction, joint movement, or even the sense of holding an object in their hand [22,23]. This method is most effective when tailored to the individual’s specific deficits, progressively increasing the complexity of imagined activities as their recovery advances [24,25]. Technology-assisted guided imagery has made great progress, including virtual reality, BCIs, and robots to improve motor rehabilitation [26,27,28,29,30,31,32]. BCIs give real-time feedback, reinforcing sensorimotor pathways, whereas robotic-assisted treatment enhances visualization by allowing for regulated, repeated motions, bridging the gap between mental rehearsal and execution [33,34,35,36,37]. Similarly, neurophysiological and neuromodulation approaches aid in the assessment and enhancement of neuroplasticity. Electroencephalography (EEG) and functional magnetic resonance imaging (fMRI) monitor brain activity changes [38], while transcranial magnetic stimulation (TMS) and transcranial direct current stimulation (tDCS) regulate cortical excitability, therefore maximizing neuronal reconfiguration [39,40]. Together, these technologies improve rehabilitation tactics by providing individualized, data-driven approaches to stroke recovery. MI is a specialized use of guided imagery in neurorehabilitation that focuses on mentally practicing certain motor activities in order to enhance functionality [41]. Motor imagery training (MIT) is especially useful for stroke survivors who have limited mobility because it activates the sensorimotor cortex without needing actual movement [42]. MI protocols for stroke recovery often include planned exercises in which patients envision themselves doing motions like gripping an item, extending their arm, or walking [43]. These exercises can be supplemented with kinesthetic imaging, in which participants mentally feel the sensation of movement, or visual imagery, in which they picture themselves completing the activity from a first- or third-person perspective [44,45]. When combined with other rehabilitation approaches (such as mirror therapy, robotic-assisted training, or neuromodulation), MI can dramatically speed up recovery by strengthening the neural circuits responsible for motor control [46]. Although growing evidence supports the use of guided imagery in stroke rehabilitation, its precise impact on motor recovery remains unclear. Significant gaps persist in understanding the most effective ways to deliver these interventions, including whether guided imagery should be applied alone or in combination with other rehabilitation approaches such as physical therapy, neuromodulation, or virtual reality training, and under what specific conditions it yields the greatest benefits. Additionally, key parameters such as the optimal frequency, intensity, and duration of guided imagery sessions have not been systematically established, making it difficult to standardize treatment protocols and compare findings across studies. The heterogeneity in outcome measures, ranging from functional motor assessments to neurophysiological markers and patient-reported experiences, further complicates the ability to draw definitive conclusions about its clinical efficacy.

This systematic review seeks to fill these gaps by compiling results solely from RCTs, offering a more thorough assessment of the efficacy of guided imagery for motor rehabilitation in individuals who have had a stroke. This review aims to pinpoint best practices for implementing guided imagery by thoroughly examining methodological differences, intervention protocols, and reported results, while also emphasizing areas that need more investigation and providing evidence-based suggestions for its incorporation into regular stroke rehabilitation programs. The rationale behind this review stems from the increasing recognition of neuroplasticity as a key mechanism in post-stroke motor recovery. Traditional rehabilitation approaches primarily rely on physical movement to stimulate motor pathways, yet many stroke patients experience significant mobility restrictions that limit their ability to engage in active training. Guided imagery offers a non-invasive, accessible alternative that can stimulate sensorimotor circuits even in the absence of overt movement, potentially accelerating functional recovery. This review is grounded in the Motor Simulation Theory and the Hebbian Theory of neuroplasticity [47,48]. The first one suggests that mentally rehearsing movements engages neural networks in the same way as physical execution, reinforcing motor representations and facilitating skill acquisition. Meanwhile, Hebbian plasticity emphasizes the principle that “neurons that fire together, wire together”, highlighting how repeated activation of sensorimotor pathways (whether through actual movement or mental practice) can strengthen synaptic connections. By integrating these frameworks, this review will assess how guided imagery techniques leverage neuroplastic mechanisms to enhance motor function, providing insights into their role as a complementary rehabilitation strategy. To further illustrate the role of guided imagery in stroke rehabilitation, Table 1 provides a detailed comparison of key techniques, their underlying neural mechanisms, application contexts, and associated neurophysiological measurements that support their therapeutic effectiveness [49,50,51,52,53].

## 2. Materials and Methods

### 2.1. Search Strategy

This systematic review employs a structured approach without applying a specific search period to ensure a comprehensive exploration of the available evidence on guided imagery techniques in stroke motor rehabilitation. By not restricting the timeframe, the review aims to capture all relevant studies, regardless of publication date, that contribute to understanding the evolution of research and advancements in this field. This approach ensures a broader perspective while maintaining focus on the most recent and applicable findings. We performed an extensive literature search using the PubMed, Web of Science, Embase, EBSCOhost, and Scopus databases, utilizing the keywords (All Fields: “Guided Imagery”) AND (All Fields: “Stroke”) AND (All Fields: “Motor Rehabilitation”) from 15 November 2024 to 9 January 2025. These databases were carefully selected to cover the broadest possible range of peer-reviewed literature in the fields relevant to this review. PubMed was selected for its deep representation of biomedical research, particularly its rich indexing of neurological disorders and rehabilitation research. Web of Science was considered for its multidisciplinary coverage and its citation tracking functionality, which allows identification of seminal works in the field. Embase was selected for its long-standing reputation for providing broad coverage of clinical and pharmacological studies, particularly in neurorehabilitation studies. EBSCOhost made a range of specialist health sciences databases available. Additionally, Scopus was selected for its broad multidisciplinary coverage and sophisticated citation analysis capabilities that help reflect the diversity of studies included in the retrieved field. Only RCTs involving adults and published in English were included in this review. By using these databases, we sought to maximize the robustness and therefore validity of our strategy, reduce the possibility of missing important studies, and allow for the inclusion of high-quality, diverse evidence.

#### 2.1.1. Data Extraction

**Figure 1:**
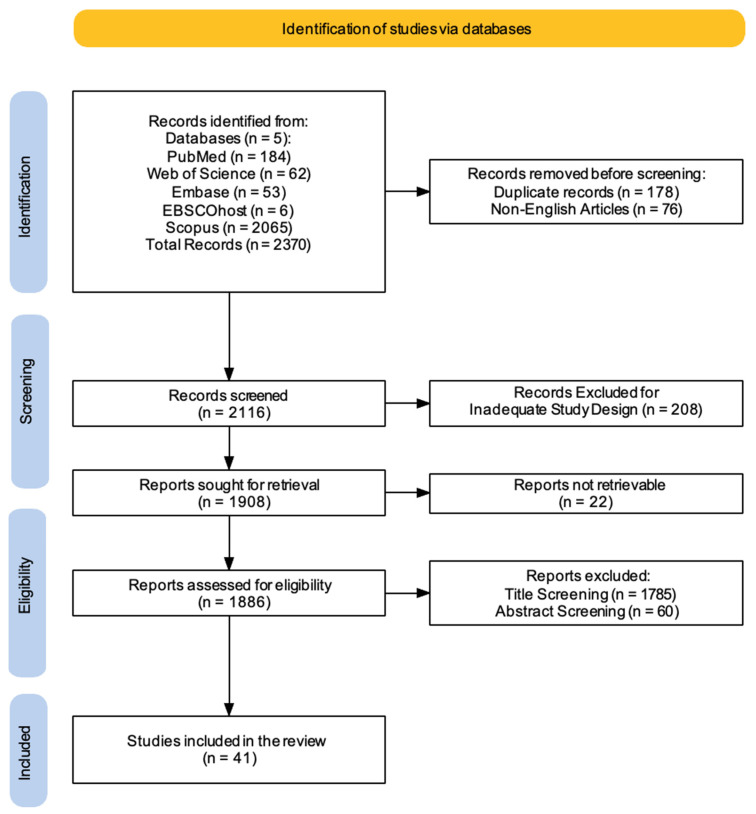
PRISMA 2020 flow diagram of evaluated studies.

Two reviewers (AC, AM) performed independent searches to improve transparency and accuracy in locating pertinent studies. The search strategy was iteratively refined by testing different combinations of keywords, Boolean operators, and controlled vocabulary (e.g., MeSH terms) to maximize sensitivity and specificity. The PRISMA flowchart was employed to depict the process (identification, screening, eligibility, and inclusion) for choosing relevant studies, as shown in Figure 1 [54]. The Cochrane Risk of Bias (RoB 2) framework was applied to assess bias risk in the RCTs included in this review. A detailed protocol for assessing bias, aligned with the Cochrane Handbook for Systematic Reviews of Interventions, was followed to maintain methodological rigor. Additionally, two researchers (AC, AM) screened all articles based on titles, abstracts, and full texts, conducting independent data extraction, article gathering, and cross-validation to minimize bias risks (e.g., missing results bias, publication bias, time lag bias, language bias). The gathered data included study design, sample size, characteristics of participants, types of strokes, specifics of the guided and MI intervention, duration, assessed outcomes, and findings. The researchers (AC, AM) reviewed complete text articles considered suitable for the study, and if there were disagreements regarding the inclusion and exclusion criteria, a final decision was reached by a third researcher (RSC). Discrepancies between reviewers during the screening or data extraction process were also resolved through discussion, with unresolved cases adjudicated by a third reviewer (RSC). Additionally, the concordance between the two evaluators (AC and AM) was evaluated through the kappa statistic. The kappa score, which has a recognized threshold for significant agreement established at >0.61, was understood to indicate substantial alignment among the reviewers. This standard guarantees a strong assessment of inter-rater reliability, highlighting the attainment of a significant degree of consensus in the data extraction procedure. Data extraction and organization were facilitated using Microsoft Excel, which streamlined the process and minimized human error. The software allowed for efficient management of large datasets, enabling reviewers to systematically record study characteristics, risk of bias assessments, and outcome data. Custom extraction sheets were designed within the software to ensure consistency and adherence to the predefined inclusion/exclusion criteria. Additionally, the software provided features such as tagging, filtering, and sorting, which facilitated the resolution of discrepancies and expedited the cross-validation process. The compilation of articles was subsequently refined for relevance, assessed, and summarized, with main topics highlighted from the summary according to the inclusion/exclusion standards. This systematic review has been registered on Open OSF under the following number: DOI 10.17605/OSF.IO/3D7MF.

#### 2.1.2. Data Synthesis

Data synthesis was conducted using narrative methods and quantitative analysis to tackle the variety of guided and MI interventions and types of strokes included. This method allowed us to identify key themes, commonalities, and differences across the research landscape. Effect sizes and evidence certainty from diverse studies reflecting various interventions and stroke populations were reported. Studies were grouped based on intervention types, patient characteristics, and reported outcomes to highlight consistencies and discrepancies. Throughout the synthesis process, the inclusion of a multidisciplinary team ensured a balanced interpretation of the data. Regular discussions and consensus meetings among reviewers helped mitigate potential biases in qualitative assessments and ensured consistency in categorizing and interpreting outcomes. This integrated approach combined qualitative insights with quantitative precision, offering a holistic understanding of the research landscape while addressing the complexities inherent in the studies.

### 2.2. PICO Evaluation

We applied the PICO model (Population, Intervention, Comparison, Outcome) to create our search terms.

The target population consists of adults who have had a stroke. The intervention being studied is guided MI, a technique that includes mentally imitating certain motor actions to improve brain plasticity and motor recovery. This will be compared against traditional motor rehabilitation treatments, such as physical therapy alone or no intervention at all, to establish its relative efficacy. The key outcomes of interest are gains in motor function, mobility, and overall recovery rates, while secondary goals include quality of life, patient adherence to therapy, and cognitive engagement throughout rehabilitation.

### 2.3. Inclusion Criteria

The inclusion criteria for this systematic review focused on identifying research studies that explored the impact of guided imagery techniques, with an emphasis on MI, on motor rehabilitation outcomes in stroke patients. To be eligible, studies needed to investigate guided imagery interventions and assess their effects on motor recovery using validated clinical, neurophysiological, or functional measures. Included articles were required to provide a clear description of the guided imagery protocols, including their structure, duration, and delivery methods, along with measurable outcomes related to motor function, mobility, or recovery. Included studies targeted adult participants (≥18 years) diagnosed with stroke and presenting motor impairments. The review specifically considered guided imagery techniques delivered either through technological devices, such as BCIs and robotic devices, or guided by a human operator, such as a clinician or therapist. The review included primary research encompassing only RCTs. This decision was based on the need to ensure a high level of evidence and minimize the risk of bias when assessing the impact of guided imagery techniques on motor rehabilitation outcomes in stroke patients. RCTs are considered the gold standard for evaluating the efficacy of interventions due to their rigorous methodology, which includes randomization and control groups to isolate the effects of the intervention. Furthermore, to ensure a comprehensive and reliable analysis, only full-text articles published in English were included.

### 2.4. Exclusion Criteria

To maintain the review’s focus on the impact of guided imagery techniques on motor rehabilitation outcomes in stroke patients, several exclusion criteria were established. Studies were excluded if they investigated interventions unrelated to guided imagery, such as pharmacological approaches, or general physical rehabilitation where guided imagery was not the central intervention. Research targeting populations other than stroke patients, including studies involving mixed neurological conditions without a distinct analysis for stroke participants, was also excluded. Additionally, studies involving children or adolescents under 18 years of age, or those that did not clearly define motor impairments linked to strokes, were omitted to ensure relevance to the review’s objectives. Articles that failed to provide adequate detail on the structure, duration, or delivery of guided imagery protocols, or that did not use validated clinical, neurophysiological, or functional measures to assess outcomes, were excluded. Only RCTs were considered, meaning non-randomized studies, feasibility studies, observational research, longitudinal studies, retrospective studies, and study protocols were excluded to uphold methodological rigor and allow for reliable causal inferences. Reviews, theoretical papers, or meta-analyses were not included, as the review aimed to synthesize primary experimental evidence. Finally, studies published in languages other than English, or available only as abstracts, posters, or conference proceedings, were excluded due to the lack of comprehensive data. Research involving animal models or preclinical studies was also omitted, as the review’s focus was on clinical applications in human populations. This careful selection process ensured that the included studies provided robust, high-quality evidence relevant to stroke rehabilitation. Table 2 provides an overview of the methodology employed for this systematic review.

## 3. Results

A comprehensive literature search was carried out using five electronic databases. This initial search yielded a total of 2370 records. Before formal screening commenced, 178 duplicate records were identified and removed, along with 76 non-English articles. This left 2116 records for title and abstract screening. Following this initial screening phase, 208 records were excluded based on inadequate study design, leaving 1908 reports for full-text retrieval. However, despite efforts to obtain these articles (e.g., emailing corresponding authors, consulting libraries, exploring open-access sources, checking institutional resources, and using research networks), 22 reports could not be retrieved. The remaining 1886 reports underwent a thorough assessment for eligibility. During this stage, a further 1785 reports were excluded based on title screening, and an additional 60 were excluded after abstract screening. This rigorous selection process resulted in a final set of 41 studies that met the pre-defined inclusion criteria and were therefore included in this review (Figure 1).

### 3.1. Quality of Included Studies—Risk of Bias

To ensure a thorough evaluation of methodological quality, we conducted a detailed assessment of bias risk using established and recognized tools specifically designed for the study design of the included papers [55,56,57,58,59,60,61,62,63,64,65,66,67,68,69,70,71,72,73,74,75,76,77,78,79,80,81,82,83,84,85,86,87,88,89,90,91,92,93,94,95]. This systematic approach provided a robust framework for identifying potential limitations and inconsistencies in research methods, enabling a transparent and reliable comprehension of the findings within the broader context of evidence.

#### Cochrane Risk of Bias Tool for Randomized Trials (RoB 2)

**Figure 2:**
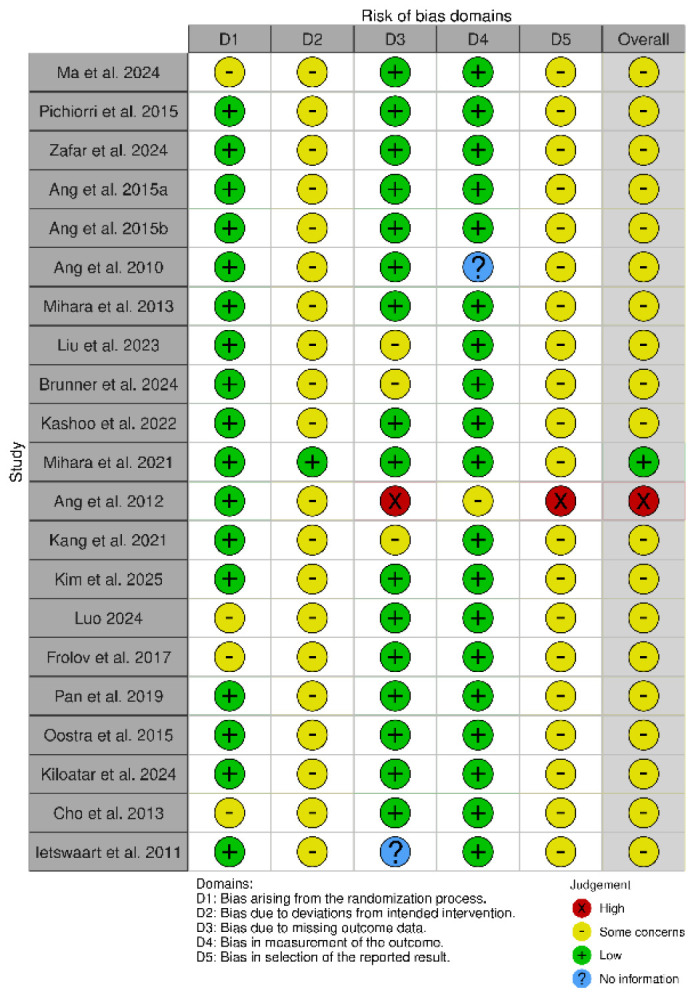
Risk of Bias (RoB) of included RCT studies [55,56,57,58,59,60,61,62,63,64,65,66,67,68,69,70,71,72,73,74,75].

**Figure 3:**
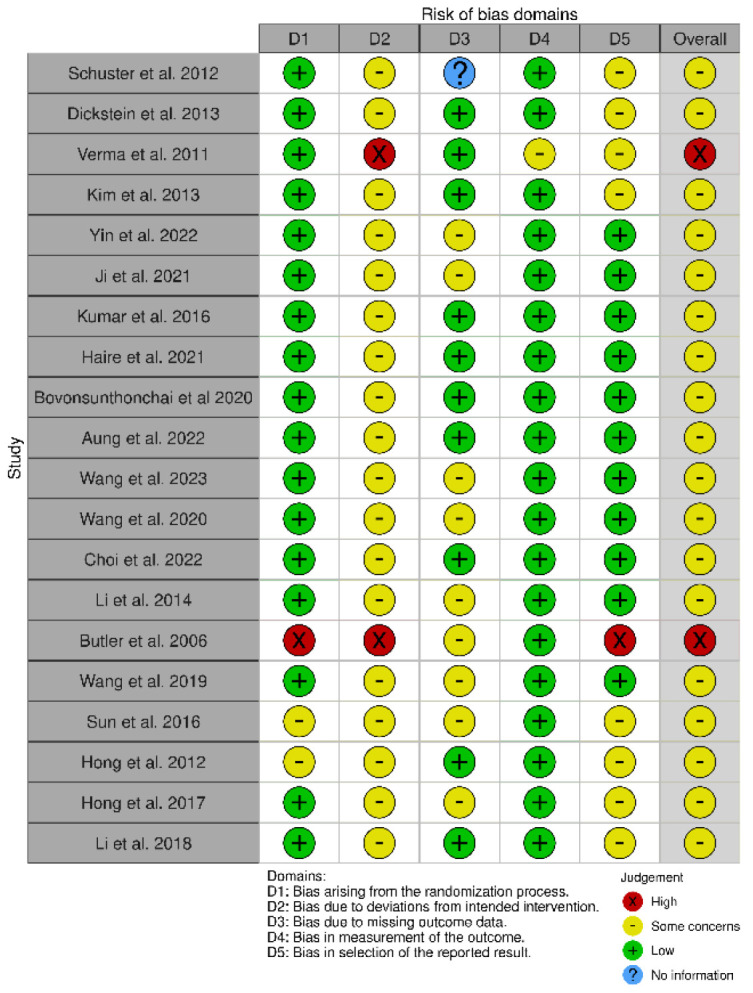
Risk of Bias (RoB) of included RCT studies [76,77,78,79,80,81,82,83,84,85,86,87,88,89,90,91,92,93,94,95].

All forty-one studies were RCTs [55,56,57,58,59,60,61,62,63,64,65,66,67,68,69,70,71,72,73,74,75,76,77,78,79,80,81,82,83,84,85,86,87,88,89,90,91,92,93,94,95]. We used the updated Cochrane Risk of Bias (RoB 2) tool, which covers five domains: (i) bias arising from the randomization process, (ii) bias due to deviations from the intended intervention, (iii) bias due to missing data on the results, (iv) bias in the measurement of the outcome, and (v) bias in the selection of the reported result (Figure 2 and Figure 3) [96].

The RoB 2 assessment revealed that most studies fell into the “some concerns” category for overall risk of bias, with only one study (Mihara et al. [65]) achieving a “low risk” rating across all domains. A notable trend emerged, with studies such as Ma et al. [53], Pichiorri et al. [56], Zafar et al. [57], and Ang et al. [58,59] consistently displaying “some concerns” in key domains, particularly related to deviations from intended interventions (D2) and selective reporting (D5). This pattern suggests recurring methodological limitations that may affect the reliability of their findings. Several common issues were observed across studies. Allocation concealment and randomization reporting were often insufficiently detailed, as seen in Luo [69] and Frolov et al. [70], increasing the risk of selection bias. Similarly, partial or poorly implemented blinding, particularly in studies relying on subjective outcome measures, was evident in Liu et al. [62] and Brunner et al. [63], raising concerns about performance and detection bias. The handling of missing data was another frequent challenge, with studies such as Wang et al. [86] and Wang et al. [87] providing limited transparency regarding their data management strategies. In certain cases, the risk of bias was more pronounced. Ang et al. [66] and Verma et al. [78] demonstrated high risk in multiple domains, including performance bias and selective outcome reporting, which significantly undermines the credibility of their conclusions. Butler et al. [90] also exhibited high risk across several domains, particularly in deviations from intended interventions and selective reporting, reflecting broader methodological concerns in early rehabilitation research. Interestingly, even among the stronger studies, concerns remained regarding selective reporting (D5). While Mihara et al. [65] demonstrated the most robust methodological rigor, studies such as Kang et al. [67] and Kim et al. [68] still exhibited “some concerns” in this domain, underscoring the need for improved transparency in reporting research outcomes. These findings carry significant implications for interpreting the existing body of evidence. The recognized methodological constraints, especially those concerning randomization and blinding, greatly affect how we interpret our results. Ambiguous or insufficient randomization methods could result in selection bias, possibly causing disparities between intervention groups that might influence study results. This constraint diminishes the certainty in assigning the observed effects exclusively to the interventions being studied. Likewise, insufficient blinding, particularly in research employing subjective outcome metrics, creates issues related to performance and detection bias. Expectations of participants and assessors can affect outcome evaluations, possibly exaggerating treatment effects. These biases together diminish the confidence in the evidence, complicating the assessment of the actual effectiveness of interventions. Thus, although our synthesis offers important perspectives on existing research trends, prudence is advisable when extending results to wider clinical applications. Tackling these methodological weaknesses in upcoming trials will be essential for reinforcing the evidence foundation and guaranteeing more dependable conclusions about the efficacy of neurorehabilitation approaches.

### 3.2. Synthesis of Evidence

All the 41 included studies investigated the application of guided imagery techniques, including MI for motor rehabilitation in strokes (see Table 3). Looking at the age ranges of participants, we found a normal distribution for stroke research, comprising middle-aged and older persons, with mean ages ranging from 50 to 70 years old in most studies. In evaluating the sample sizes of both the treatment and control groups, the studies showed a certain level of consistency. Although there was some fluctuation, the mean number of participants in both the treatment and control groups was roughly 20. This indicates that researchers usually sought comparable group sizes to ensure balance and consistency among interventions. Nonetheless, it is crucial to recognize that certain studies had smaller sample sizes, whereas others had larger ones, which could influence the statistical power of each study. Concerning sex distribution, the research typically indicated a larger share of male participants. On average, around 65% of the individuals in both the treatment and control groups were male. This higher incidence in males aligns with noted trends in stroke demographics, indicating that men are statistically more prone to having strokes compared to women. The duration of the intervention varied. Some studies used relatively brief treatments (a few weeks), whereas others used lengthier programs (many months). Furthermore, the studies used a variety of methods to evaluate upper extremity function, including both impairment-based measures like the Fugl–Meyer Assessment (FMA) and activity-based measures like the Action Research Arm Test (ARAT) and the Wolf Motor Function Test (WMFT). Furthermore, the examined research provided a mixed picture in terms of impact sizes and evidentiary certainty. Some studies suggested moderate to substantial benefits, particularly when guided imagery was paired with other treatments. The level of confidence in evidence also varies. Many studies are characterized as having “moderate” assurance of evidence. This frequently indicates research design flaws, such as limited sample numbers, a lack of blinding, or ambiguous randomization processes. The studies frequently integrate guided imagery with traditional physical therapy interventions, such as range of motion exercises, task-specific training, and constraint-induced movement therapy. This combination likely creates a synergistic effect, where guided imagery amplifies the benefits of conventional rehabilitation techniques. Essentially, guided imagery seems to prime the brain and nervous system for motor learning and functional improvement, making other therapies more effective.

To further investigate all the obtained results, we sequentially summarized the primary insights regarding how guided imagery techniques impact motor stroke rehabilitation [55,56,57,58,59,60,61,62,63,64,65,66,67,68,69,70,71,72,73,74,75,76,77,78,79,80,81,82,83,84,85,86,87,88,89,90,91,92,93,94,95]. The first fifteen studies analyzed MI in stroke rehabilitation [57,72,73,74,75,76,77,78,79,80,81,82,83,84,85]; the other eleven articles investigated the combined use of MI and BCI [55,56,58,59,60,62,63,66,68,69,70]. Five studies investigated the impact of neurofeedback and non-invasive brain stimulation techniques combined with MI [61,64,65,67,71], while the last ten papers explored the neuroplastic effects of MI [86,87,88,89,90,91,92,93,94,95].

#### Safety and Adverse Events

Across the multiple RCTs investigating MI for stroke rehabilitation, MI treatments, whether used alone or in combination with other therapies, consistently show a positive safety profile. No serious adverse events were directly attributable to MI in any of the reviewed studies. While some research incorporated combined interventions like MI with robotic therapy, BCIs, functional electrical stimulation (FES), or non-invasive brain stimulation (e.g., tDCS, TMS), these additions did not introduce significant safety concerns beyond the known risks associated with the individual components themselves. The most commonly reported “adverse events” were related to the underlying condition of the stroke (e.g., falls, pain, fatigue, muscle soreness, or discomfort related to other concurrent therapies), rather than the MI intervention. Generally, MI protocols, including those focused on gait, upper limb function, and other motor recovery domains, were well tolerated by participants. Although detailed safety data may not have been the primary focus of every study, the accumulated evidence strongly suggests that MI-based rehabilitation is a safe and well-tolerated approach for stroke patients. No consistent pattern of adverse events related specifically to MI emerged from the analyzed literature.

### 3.3. Motor Imagery in Stroke Rehabilitation: Gait, Upper Limb Function, and Beyond

A growing body of research highlights the potential of MI as a valuable tool in stroke rehabilitation, with numerous studies exploring its impact on various aspects of motor recovery. A first RCT (n = 44 stroke patients, n = 27 healthy controls) looked at the effect of MIT on gait recovery in sub-acute stroke patients. After six weeks, the MIT group, who received normal therapy as well as 30 min daily MIT sessions, improved their gait velocity much more than the muscular relaxation group. While all groups improved lower extremity function, the MIT group had greater increases in kinesthetic MI capacity [72]. Another RCT (n = 10 MI, n = 15 action observation, n = 11 action execution) explored the impact of MI on the autonomic nervous system (ANS) during walking in chronic stroke patients. While action execution significantly increased heart rate and decreased the mean RR interval, indicating ANS activation, MI did not elicit significant changes in ANS activity [73]. Cho et al. examined the impact of MIT combined with gait training on balance and gait (n = 28 chronic stroke patients). The experimental group (n = 15) received both MIT and gait training, while the control group (n = 13) received only gait training. After six weeks, the MIT group demonstrated significantly greater improvements in functional reach, timed up-and-go, 10 m walk, and FMA scores compared to the control group [74]. An RCT investigated the effectiveness of MIT on upper limb motor recovery (n = 121 stroke patients). Participants were assigned to either an MIT group, an attention-placebo control group, or a normal care control group. While all groups showed improvement, no significant differences in recovery (ARAT score, grip strength, manual dexterity) were found between the MIT group and the control groups after four weeks of training [75]. Schuster and colleagues investigated the impact of embedded or added MIT on a complex motor task (“Going down, laying on the floor, and getting up again”) (n = 41 stroke patients). While all groups (embedded MI, added MI, control) improved their performance time on the task after a two-week intervention, no significant differences were found between the groups [76]. Dickstein and his team examined the impact of integrated MI on ambulation (n = 23 community-dwelling stroke survivors). Participants received either integrated MI or a control treatment (upper extremity exercises) for four weeks, with the control group crossing over to integrated MI in the second phase. Integrated MI significantly improved indoor walking speed compared to the control treatment, with gains maintained at follow-up. However, no significant improvements were observed in community ambulation or fall-related self-efficacy [77]. Verma et al. investigated the impact of task-oriented circuit class training (TOCCT) combined with MI on gait rehabilitation (n = 30 subacute stroke patients). The experimental group (n = 15) received MI followed by TOCCT, while the control group (n = 15) received conventional Bobath-based therapy. After two weeks, the MI + TOCCT group showed significantly greater improvements in functional ambulation, gait speed, endurance, and gait quality compared to the control group [78]. Kim and colleagues compared action observation training (AOT), MIT, and physical training on dynamic balance and gait (n = 27 chronic stroke patients). Participants received 4 weeks of training. AOT showed significant improvements compared to physical training in the timed up-and-go (TUG) test, gait speed, cadence, and single limb support. While both AOT and MIT improved dynamic balance and gait, AOT demonstrated superior gains, suggesting that visually learning movements enhances motor recovery more effectively than MI alone [79]. Yin and his team explored the impact of MIT combined with conventional rehabilitation on lower limb motor recovery (n = 32 chronic stroke patients). The experimental group received MIT in addition to standard care, while the control group received only standard care. After six weeks, the MIT group showed significantly greater improvements in lower extremity motor function, balance, and activities of daily living compared to the control group [80]. Another RCT investigated the impact of a home-based graded motor imagery (GMI) program on upper limb motor function (n = 37 chronic stroke patients). The GMI group received implicit MI, explicit MI, and mirror therapy, while the control group received conventional therapy only. After 8 weeks, the GMI group showed significantly greater improvement in upper limb motor function compared to the control group, suggesting that GMI can be a useful adjunct to conventional rehabilitation for improving upper extremity function post-stroke [81]. Kumar and colleagues examined the effect of adding MI to task-oriented training on paretic lower extremity muscle strength and gait performance (n = 40 ambulant stroke patients). The experimental group received MI training with audio guidance in addition to task-oriented exercises, while the control group received task-oriented exercises alone. After three weeks, the MI group demonstrated significantly greater improvements in hip flexor/extensor strength, knee extensor strength, ankle dorsiflexor strength, and gait speed compared to the control group [82]. Haire et al. investigated the effects of therapeutic instrumental music performance (TIMP) with and without MI on cognitive and affective outcomes (n = 30 chronic stroke patients). Participants were assigned to TIMP only, TIMP with cued MI (TIMP + cuedMI), or TIMP with MI (TIMP + MI) groups. The TIMP + MI group showed significant improvement in cognitive flexibility, while the TIMP + cuedMI group demonstrated significant positive affect changes [83]. An RCT investigated the effects of MI combined with structured progressive circuit class therapy (SPCCT) on gait and muscle strength (n = 40 stroke patients). The experimental group received MI training before SPCCT, while the control group received health education before SPCCT. After 4 weeks, the MI group showed significantly greater improvements in gait speed, cadence, step length, and hip/knee extensor strength compared to the control group [84]. Aung et al. investigated the impact of MI combined with SPCCT on functional mobility (n = 40 post-stroke patients). The experimental group received MI before SPCCT, while the control group received health education before SPCCT. After 4 weeks, the MI group demonstrated significantly greater improvements in step test scores (affected and unaffected limbs), 6 min walk test distance, and TUG test performance compared to the control group [85]. An RCT involved 29 stroke patients (15 in the experimental group, 14 in the control group) who completed an 8-week intervention. The experimental group received MI along with traditional motor rehabilitation, while the control group received only the latter. Results showed significant improvements in mobility, balance, and fall risk in the MI group [57]. The findings underscore the potential of MI as a valuable adjunct to conventional rehabilitation approaches for individuals seeking to regain motor function after stroke.

### 3.4. Brain–Computer Interface and Motor Imagery for Stroke Rehabilitation: An Overview of Clinical Studies

Several RCTs have investigated the efficacy of MI-based BCI rehabilitation for stroke patients, with varying results. A first RCT investigated the effects of MI-based BCI rehabilitation on upper limb motor function and brain activation (n = 40 chronic stroke patients). The experimental group received MI-BCI training combined with conventional therapy, while the control group received conventional therapy alone. Post-intervention, the MI-BCI group showed significant improvement in upper extremity motor function compared to the control group, accompanied by increased activation in motor-related brain regions during motor execution and imagery tasks [55]. Pichiorri et al. investigated the impact of BCI-supported MI training on upper limb motor recovery (n = 28 subacute stroke patients). Participants were assigned to either a BCI-supported MI group or a control group receiving MI training alone. After 1 month, the BCI group showed a trend towards greater improvement in the primary outcome measure, the arm section of the FMA, compared to the control group, suggesting that the added BCI assistance may enhance the benefits of MI for motor rehabilitation [56]. Ang and his team explored the impact of BCI-based MI training combined with robotic therapy on upper limb motor function (n = 26 chronic stroke patients). The experimental group received BCI-MI training with robotic feedback, while the control group received robotic therapy alone. After 4 weeks, both groups showed significant improvements in upper limb motor function, with no significant difference between groups, suggesting that BCI-MI training may offer comparable benefits to robotic therapy with fewer repetitions [58]. Another RCT investigated the effects of tDCS combined with MI-based BCI and robotic therapy on upper limb motor recovery (n = 19 chronic stroke patients). Participants were assigned to either a tDCS with MI-BCI and robotic therapy group or a sham-tDCS group. While both groups showed motor function improvements after 2 weeks of intervention, no significant difference was observed between the groups. However, the tDCS group demonstrated significantly higher online accuracy in MI-BCI performance and a higher event-related desynchronization laterality coefficient, suggesting that tDCS may facilitate MI in chronic stroke patients [59]. Another paper investigated the ability to operate an EEG-based MI-BCI and the efficacy of MI-BCI with robotic feedback on motor recovery (n = 54 BCI-naïve hemiparetic stroke patients). Results showed that 89% of patients could operate the MI-BCI better than chance, with no correlation between MI-BCI ability and baseline motor function. A subset of these patients (n = 26) was then randomized into either the MI-BCI with robotic feedback group or the robotic rehabilitation group. While the robotic rehabilitation group showed earlier motor function improvements, both groups achieved significant improvements post-rehabilitation and at 2-month follow-up, with no significant difference between groups [60]. Liu and colleagues investigated the effects of MI-based BCI training combined with conventional rehabilitation on upper limb motor function and attention (n = 60 subacute stroke patients). The experimental group received MI-BCI training with FES in addition to conventional therapy, while the control group received conventional therapy alone. After 3 weeks, the MI-BCI group showed significant improvements in upper limb motor function and attention compared to the control group [62]. Brunner and his team investigated the effects of BCI training on upper limb motor recovery (n = 40 acute stroke patients). The experimental group received BCI training with FES as part of their standard upper limb rehabilitation, while the control group received standard rehabilitation alone. After 3 months, both groups showed improvements in upper limb motor function, but no significant difference was found between the groups. However, EEG analysis in the BCI group revealed significant changes in brain activity associated with MI [63]. A clinical trial (n = 19 BCI-naïve hemiparetic stroke patients recruited; 5 completed 10 rehabilitation sessions) is investigating the effects of tDCS combined with MI-based BCI and robotic feedback on upper limb stroke rehabilitation. Preliminary results from the completed sessions show no significant difference in the online accuracy of MI detection between the tDCS and sham-tDCS groups. However, offline analysis suggests that the tDCS group may exhibit higher accuracy in classifying MI compared to the sham group, hinting at a potential facilitating effect of tDCS on MI, although this requires further investigation with a larger sample size. The study also found that 68% of the stroke patients screened were able to operate the MI-BCI better than chance [66]. Kim et al. investigated the effects of MI-contingent BCI training on upper limb function and neural connectivity (n = 25 chronic stroke patients). The experimental group received BCI training with FES contingent on successful MI, while the control group received FES independent of MI. After 4 weeks, the MI-contingent group showed significant improvements in wrist extensor muscle strength compared to the control group. Additionally, this group exhibited increased neural connectivity in brain regions associated with motor control [68]. Another RCT investigated the effects of BCI rehabilitation combined with conventional rehabilitation on lower limb motor function (n = 64 acute ischemic stroke patients). The experimental group received BCI-controlled electrical stimulation in addition to conventional rehabilitation, while the control group received conventional rehabilitation alone. After 2 weeks, the experimental group showed significantly greater improvements in lower limb motor function, walking ability, and activities of daily living compared to the control group [69]. Frolov and his team investigated the effects of BCI training combined with a hand exoskeleton on upper limb motor recovery (n = 74 subacute and chronic stroke patients). The experimental group received BCI-controlled exoskeleton training, where the exoskeleton’s movements were contingent on the patient’s MI, while the control group received passive exoskeleton movements. After 10 sessions, the BCI group showed significant improvements in hand function (grasp, pinch, gross movements) compared to the control group, highlighting the role of MI in driving motor recovery through BCI-mediated feedback. While overall arm function improved in both groups, the BCI group demonstrated greater gains in hand-specific tasks [70]. Collectively, these studies suggest that MI-BCI can be a valuable tool for stroke rehabilitation, but its optimal implementation and comparative effectiveness relative to other therapies require additional investigation.

### 3.5. The Impact of Augmented Motor Imagery on Post-Stroke Rehabilitation: Exploring Neurofeedback and Non-Invasive Brain Stimulation Techniques

A variety of studies have explored the use of neurofeedback and non-invasive brain stimulation techniques in conjunction with MI to enhance upper limb motor recovery in stroke patients. A pilot study investigated the effects of MI with near-infrared spectroscopy (NIRS) neurofeedback on upper limb motor recovery in 20 subcortical stroke patients. Participants were randomized into two groups: one receiving real-time feedback of their brain activity during MI (REAL group) and a control group receiving sham feedback (SHAM group). After a two-week intervention (three sessions/week), the REAL group showed significant improvements in hand/finger function (FMA) compared to the SHAM group, suggesting that NIRS-guided MI enhances motor recovery by increasing activation in the ipsilesional premotor area [61]. Kasho et al. investigated the effects of combining visual motor imagery (VMI) with tDCS on upper limb motor recovery in 64 chronic stroke patients. Participants were assigned to either a VMI-plus-tDCS group or a VMI-only control group. Results showed significant improvements in both the FMA and the ARAT scores in the combined VM” and’tDCS group compared to the control group, indicating that the addition of VM” and’tDCS enhances motor rehabilitation outcomes. Specifically, the combined therapy group demonstrated clinically meaningful improvements in upper limb function [63]. Mihara and colleagues (n = 54) investigated the impact of functional near-infrared spectroscopy (fNIRS) neurofeedback combined with gait and balance-related MI on post-stroke gait disturbances. The real feedback group, receiving genuine neurofeedback of their supplementary motor area (SMA) activity during MI, showed significantly greater improvement in the TUG test compared to the sham feedback group [65]. Kang et al. (n = 17) investigated the effects of combining low-frequency repetitive transcranial magnetic stimulation (LF-rTMS), audio-based MI, and electrical stimulation (ES) on upper extremity motor recovery in subacute stroke patients. The experimental group, receiving LF-rTMS, audio-based MI, and active ES, showed significantly greater improvement in the FMA scores compared to the control group, which received LF-rTMS, audio-based MI, and sham ES [67]. One last RCT (n = 42) explored the effects of combining LF-rTMS with audio-based MI on upper limb motor recovery in chronic stroke patients. The experimental group, receiving LF-rTMS and MI, demonstrated significantly greater improvements in the WMFT, FMA, MBI, and Box and Block Test compared to the control group, which received LF-rTMS and audiotape-led relaxation [71]. The findings suggest that integrating MI with techniques like NIRS, tDCS, and rTMS may enhance motor recovery beyond what is achievable with MI alone, although further research is needed to optimize these interventions.

### 3.6. Neuroplastic Effects of Motor Imagery Training in Stroke Rehabilitation: Evidence from RCTs

Recent studies have explored the efficacy of MI-based interventions in improving upper limb function and promoting cortical reorganization in stroke patients. An RCT (n = 39 patients) investigated the effects of MIT combined with conventional rehabilitation on upper limb motor recovery and associated brain activity in chronic stroke patients. The MIT group showed significantly greater improvements in the upper extremity Fugl–Meyer score compared to the control group receiving only conventional rehabilitation. Task-based fMRI revealed that MIT led to decreased compensatory brain activation in the contralesional sensorimotor cortex and increased functional connectivity within the ipsilesional hemisphere, specifically between the ipsilesional M1 and putamen, correlating with clinical improvements [86]. Wang et al. examined the impact of MIT on brain activity and motor recovery in 31 stroke patients. The MIT group, receiving MIT in addition to conventional rehabilitation, showed significantly greater improvement in the FM-UL score compared to the control group. fMRI analysis revealed that MIT increased activity in the ipsilesional inferior parietal lobule (IPL) and enhanced functional connectivity between the IPL and other brain regions associated with motor planning and learning [87]. Choi et al. investigated the impact of combining AO with MI on upper extremity function and corticospinal activation in 45 subacute stroke patients. The experimental group, receiving AO with MI, showed significantly greater improvements in the FMA-UE and Motor Activity Log compared to the control group receiving AO alone. While both groups showed increased motor-evoked potentials amplitude, the AO with MI group demonstrated a significantly greater change [88]. Li et al. investigated the impact of an MI-based BCI combined with FES on upper extremity motor recovery in 15 subacute stroke patients. The BCI-FES group showed significant improvements in both FMA and ARAT scores after 8 weeks of training. Furthermore, this group exhibited stronger event-related desynchronization in the affected sensorimotor cortex, suggesting increased neural activity related to MI [89]. A pilot study explored the effects of mental practice combined with constraint-induced movement therapy (CIMT) on upper extremity motor recovery in four stroke patients. One patient receiving CIMT alone showed improvements in motor function and increased bilateral cortical activation. Another patient, receiving only mental practice, showed slight improvements in some measures but they were not clinically meaningful. Two patients receiving combined CIMT and mental practice showed improvements in motor function and mental imagery abilities, with one exhibiting focal contralateral activation in the motor cortex [90]. An RCT investigated the effects of MIT combined with conventional rehabilitation therapy (CRT) on motor recovery in 31 subcortical stroke patients. The MIT group showed significantly greater improvement in Fugl–Meyer Upper Limb scores compared to the CRT-only group. fMRI analysis revealed increased functional connectivity within specific brain regions related to MI and learning in the MIT group, along with an increased clustering coefficient, suggesting improved local information processing efficiency [91]. Sun et al. investigated the impact of MIT combined with CRT on motor recovery in 18 chronic stroke patients with severe hemiparesis. The MIT group showed significantly greater improvement in Fugl–Meyer Upper Limb scores compared to the CRT-only group after 4 weeks. fMRI analysis revealed two patterns of cortical reorganization: increased activation in the contralateral sensorimotor cortex and a focusing of activation in the same section with an increased laterality index [92]. Hong and colleagues compared the effects of mental imagery training combined with electromyography-triggered electrical stimulation (MIT-EMG) to FES alone on motor recovery in 14 stroke patients. The MIT-EMG group showed significantly greater improvement in the upper extremity component of the FMA compared to the FES group. MIT-EMG also led to increased cerebral glucose metabolism in specific brain regions associated with motor control, suggesting that MI facilitated cortical reorganization and enhanced motor recovery [93]. Another study with 19 patients investigated the combined effects of MI-BCI training and tDCS on motor recovery after stroke. While both MI-BCI with tDCS and MI-BCI with sham-tDCS groups showed improved motor function, there was no significant difference between the groups. However, the tDCS group exhibited increased white matter integrity in brain regions associated with motor control and enhanced cerebral blood flow in the sensorimotor cortex, suggesting that tDCS may have further promoted neuroplasticity [94]. One last RCT investigated the effects of MIT combined with traditional rehabilitation versus traditional rehabilitation alone on hand function in 20 stroke patients. The traditional rehabilitation group showed significantly greater improvement in ARAT and FMA scores compared to the traditional-rehabilitation-alone group after 4 weeks. Additionally, the traditional-rehabilitation group exhibited increased motor-evoked potentials amplitude and improved white matter integrity in the dorsal pathways, suggesting that MIT enhanced motor recovery by promoting neuroplasticity in these pathways [95]. The observed improvements in motor function and neural connectivity highlight the potential of MI as a complementary approach in post-stroke rehabilitation.

## 4. Discussion

MI has become a promising method in post-stroke rehabilitation, especially for enhancing gait speed, postural stability, and upper limb performance. When used alongside traditional rehabilitation, MI seems to improve motor recovery, though its effectiveness differs based on intervention intensity, patient involvement, and methodological aspects. Although certain studies indicate notable functional improvements, others propose that MI might not provide any extra advantages beyond traditional therapy, emphasizing the necessity for more standardized protocols and personalized treatment methods. Aside from motor function, MI’s effects on autonomic regulation and cognitive flexibility are still minimal when utilized alone. Nevertheless, when combined with multisensory feedback, like action observation or therapeutic music, it might enhance cognitive and emotional health. This indicates that MI’s possibilities go beyond motor recovery, affecting wider elements of neurorehabilitation. The combination of MI and BCI technology has yielded encouraging outcomes, especially in the rehabilitation of upper limbs, exhibiting significant enhancements in motor functions and cortical activation. Nonetheless, comparative research shows that MI-BCI training yields results comparable to robotic-assisted therapy, indicating that its effectiveness may rely on personal neuroplasticity, commitment, and the particular rehabilitation setting. 

### 4.1. From Theory to Practice: Integrating Mental Imagery for Improved Motor Function

Our comprehensive synthesis significantly builds upon a growing body of literature that highlights the benefits of guided imagery in stroke rehabilitation, yet it explicitly addresses a critical gap: the specific impact of these techniques on motor recovery. Although previous studies have reported overall improvements in patient outcomes, such as enhanced quality of life and reduced anxiety [97,98], few have systematically focused on quantifying the motor benefits that guided imagery can provide. In many cases, the literature has concentrated on general rehabilitation outcomes rather than delineating the precise mechanisms and efficacy in enhancing motor function [99]. Our work not only confirms the positive trends observed in earlier investigations [100] but also offers a more detailed synthesis of how guided imagery can be optimally integrated with conventional therapeutic modalities to improve motor recovery in stroke patients. The evidence consistently supports the use of MI as a valuable adjunct to traditional rehabilitation approaches, with significant improvements in key motor functions. For instance, several studies have demonstrated that MI, when incorporated into task-oriented exercises or circuit class therapies, significantly improves lower extremity strength, functional mobility, and overall gait quality, highlighting the importance of this approach in enhancing functional independence post-stroke [78,81]. Moreover, our synthesis underscores the varying effects of MIT depending on its integration into different rehabilitation strategies. In particular, the addition of MI to SPCCT has been shown to boost muscle strength, particularly in the hip, knee, and ankle regions, as well as improve gait speed and step length, resulting in more efficient ambulatory patterns [84]. This suggests that MI not only enhances motor performance in terms of voluntary movement control but also contributes to muscle strengthening, which is critical for improving functional mobility in stroke survivors. Similar improvements were observed when MI was combined with task-oriented training for paretic lower extremities, where significant enhancements in muscle strength and gait performance were recorded, particularly in patients with chronic stroke [82]. The benefits of MI are not limited to physical function; the integration of MI with therapies such as TIMP also demonstrates positive effects on cognitive and emotional outcomes, such as cognitive flexibility and affective responses [83]. This multidimensional impact of MI suggests that its role extends beyond purely motor recovery and may also contribute to enhancing quality of life by addressing emotional and cognitive aspects of rehabilitation. This approach bridges the gap by mapping out the specific delivery methods and intervention protocols that are most effective, thereby providing clinicians with actionable insights and reinforcing the notion that guided imagery can be a critical adjunct in stroke rehabilitation programs.

### 4.2. Bridging the Gap Between Intention and Execution: The Impact of BCI-Based Motor Imagery in Stroke Recovery

The incorporation of advanced technologies such as BCIs, robotics, and non-invasive brain stimulation into guided or MI protocols represents a transformative development in the field of stroke rehabilitation. BCI-based MIT leverages real-time neural feedback to dynamically engage and reinforce motor pathways, potentially accelerating the recovery process [101]. BCI-based MIT has been widely explored for its potential to enhance neuroplasticity and promote motor recovery. But how does it compare to traditional approaches? On one hand, numerous studies suggest that BCI-MI therapy can significantly improve upper limb function after a stroke, particularly in patients with severe impairments [55,56,58]. By decoding brain activity in real time, BCI provides immediate feedback, reinforcing neural pathways associated with movement. Theoretically, this should accelerate motor relearning, but does it always work in practice? Some RCTs, such as Ma et al. [55], Pichiorri et al. [56], Ang et al. [58], and Liu et al. [62], explored in this review have demonstrated superior recovery rates with BCI-MI compared to standard therapy, particularly when combined with FES or robot-assisted rehabilitation. There is compelling evidence that the integration of these instruments into these protocols could offer significant enhancements in motor performance, particularly in terms of gait dynamics and upper limb strength. This suggests that BCI does not just train the brain; it helps bridge the gap between intention and execution. However, other studies indicate no significant differences between BCI-MI and conventional therapy, leading to questions about who benefits most from these interventions [59,60,66]. Are we seeing a true neuroplastic effect, or are the improvements simply a result of increased patient engagement? These mixed findings underscore the complexity of translating neurotechnological advancements into clinically effective interventions. One critical factor to consider is inter-individual variability; not all patients exhibit the same capacity for MI, and differences in brain lesion location, cognitive function, and neural plasticity could influence the effectiveness of BCI training. This raises the question of whether BCI should be tailored to specific patient subgroups rather than being applied as a one-size-fits-all approach. Moreover, the observed improvements might not be solely attributable to neuroplastic changes. Some researchers argue that the intensive, engaging nature of BCI training itself could drive functional recovery, irrespective of the neural mechanisms involved. If this is the case, how much of the benefit comes from BCI-specific processes, and how much is due to increased patient motivation and participation? Addressing these uncertainties requires well-designed, large-scale clinical trials that not only compare BCI-MI to conventional therapy but also explore its long-term sustainability. While short-term gains have been reported, do these improvements translate into lasting functional independence, or do they plateau once therapy ends? Furthermore, cost-effectiveness and accessibility remain key barriers to widespread clinical implementation; without solutions to these challenges, BCI-based rehabilitation may remain confined to research settings rather than becoming a standard clinical tool. Thus, while the integration of BCI into MI therapy represents an exciting frontier, the current body of evidence suggests that we are not yet at a definitive paradigm shift. Instead, we are at a crucial stage of refinement and validation, where the next steps will determine whether BCI-based rehabilitation can truly revolutionize stroke recovery or remain an adjunct therapy for select cases. Future research should focus on identifying clear biomarkers for patient selection, optimizing training protocols, and ensuring real-world applicability to fully harness the potential of these technologies. Emphasis should also be placed on longitudinal studies to assess the sustainability of these improvements over time, as well as on exploring the cost-effectiveness and scalability of technology-integrated rehabilitation programs in diverse clinical settings.

### 4.3. Exploring the Therapeutic Potential of Motor Imagery in Stroke Recovery Through Neuroplastic Changes

The neuroplastic effects induced by guided imagery techniques are increasingly recognized as a cornerstone of their therapeutic potential in stroke rehabilitation. Several studies have provided robust evidence that MIT can facilitate significant neurophysiological changes, which in turn contribute to motor recovery [86,87,88,89,90,91,92,93,94,95]. For example, research by Wang et al. [87] revealed that guided imagery not only enhances activation in motor-related areas of the ipsilesional cortex but also improves functional connectivity within the motor network. Similarly, Choi et al. [88] demonstrated that combining AO with MI resulted in a more pronounced increase in motor-evoked potentials, suggesting that these interventions stimulate compensatory neural mechanisms. Li et al. [89] further corroborated these findings by showing that integrating a BCI-FES approach with MI leads to greater event-related desynchronization in affected cortical regions, thereby providing objective neurophysiological evidence of enhanced cortical reorganization. These neuroplastic adaptations are vital, as they indicate the brain’s ability to reorganize and form new neural connections in response to rehabilitation, ultimately supporting improved motor function. These findings align with the growing body of literature supporting the role of neuroplasticity in stroke recovery [102,103,104]. The enhanced functional connectivity, increased activity in motor-related areas, and improvements in motor function reported in these studies underscore the potential of MI as a robust therapeutic tool [87,88,89]. Clinical applications of these neuroplastic effects in motor rehabilitation are promising. For example, integrating MIT into conventional rehabilitation protocols could improve outcomes for patients with both chronic and subacute strokes by enhancing motor recovery and accelerating the reorganization of the sensorimotor cortex [105]. Furthermore, the use of imagery-based interventions, in combination with AO or tDCS, holds the potential to improve neural efficiency and function even in patients with severe hemiparesis, where traditional approaches may have limited efficacy. Future research should focus on leveraging advanced neuroimaging techniques, such as fMRI and diffusion tensor imaging, to further elucidate the mechanisms underlying these changes. Additionally, there is a need for studies that explore individualized guided imagery protocols based on patients’ unique neurophysiological profiles, thereby optimizing the intervention’s effectiveness and contributing to the development of personalized rehabilitation strategies that could dramatically improve long-term functional outcomes.

### 4.4. The Strength of Evidence: Methodological Challenges and Future Pathways in Guided Imagery Research

While this review encompasses extensive work focused solely on treating strokes through guided imagery, including MI, the overall confidence in the resulting evidence remains moderate, indicating a need for caution. It is still uncertain whether the support can be provided solely for the use of MI. Although trials typically indicate positive effects, especially when used alongside standard treatment, there are not enough high-quality data to recommend its exclusive use. This is in part due to the relative scarcity of trials that compare the exclusive use of MI with various forms of intervention or against the treatment/no-treatment controls. Furthermore, methodological issues such as limited sample sizes, lack of blinding, and inconsistent randomization add to the complexity of the situation. To determine the genuine effectiveness of the sole use of MI, future research must adopt more rigorous study designs, such as those involving adequately sized groups following identical protocols and those employing blinded trials. One major challenge is the inconsistency in reported benefits. Some report a moderate to considerable improvement, whereas others indicate no notable change. Several factors likely contribute to these mixed results. Firstly, the location and severity of the stroke, along with the duration following the stroke, significantly differ across studies and influence the patient response to MI. Secondly, the approach to treating MI differs greatly—some use brief and mild interventions while others implement structured and intensive therapies. Third, researchers assess motor improvement differently: some prioritize impairment-based measures (like the FMA), while others prioritize activity-based measures (such as the Action Research Arm Test). These obstacles complicate drawing study conclusions and make it challenging to align study results. Another problem is the heterogeneity of the included studies, which restricts the capacity to conduct a significant meta-analysis. In the absence of a standardized method for defining participant traits, intervention procedures, and outcome metrics, synthesizing results to offer clear clinical direction becomes difficult. Upcoming studies should strive for enhanced methodological consistency, explicitly detailing MI protocols, outlining target populations, and employing validated, standardized assessment instruments to enhance comparability between studies. Even with these obstacles, there is thrilling potential in merging guided imagery with new technologies such as BCI, neurofeedback, and non-invasive brain stimulation. These methods might improve the efficacy of MI, yet significant questions persist: What are the best parameters (frequency, duration, intensity) for combining MI with technology? In what ways can these interventions be tailored to meet the specific needs of individual patients? What are the neural processes that account for the noticed advantages? In what ways does the treatment using combined MI technology differ from MI by itself or technology by itself? What are the lasting impacts on motor skills and overall well-being? Answering these queries will be crucial for establishing evidence-based protocols that enhance the clinical effectiveness of guided imagery in stroke recovery. Although MI shows potential, its complete capability can only be unlocked through robust, well-structured research that enhances its application and pinpoints its most efficient use cases.

### 4.5. Strengths and Limitations

This systematic review presents a comprehensive and rigorous approach to understanding the effects of guided imagery rehabilitation on motor recovery among individuals with stroke. The strengths of the review are evident in its methodological design, transparency, and the thoroughness with which the search strategy was implemented. By systematically searching multiple well-regarded databases such as PubMed, Web of Science, Embase, EBSCOhost, and Scopus, the review captures a wide range of relevant studies, ensuring a diverse and high-quality representation of the field. This comprehensive approach enhances the likelihood of capturing all relevant research, thereby minimizing potential biases associated with an overly narrow scope. Another significant strength of the review lies in its rigorous assessment of the risk of bias, using established frameworks like the Cochrane Risk of Bias 2 tool for randomized trials. A notable strength of this review is its focus on the integration of guided imagery with other cutting-edge rehabilitation technologies, such as BCIs, robotic devices, and non-invasive brain stimulations. By examining how guided imagery can be combined with these technologies, the review highlights the potential for synergistic effects and more effective rehabilitation outcomes. This focus on technology integration is particularly relevant in the context of modern rehabilitation practices, where technology plays an increasingly important role. Furthermore, the review delves into the neurophysiological mechanisms underlying the benefits of guided imagery. By exploring how guided imagery can influence brain plasticity, cortical excitability, interhemispheric balance, and functional connectivity, the review provides valuable insights into the therapeutic potential of this technique. This emphasis on the neurophysiological aspects of guided imagery is crucial for understanding how it works and for developing more targeted and effective interventions. Moreover, the review emphasizes the safety of this approach, highlighting that guided imagery is a non-invasive, low-risk intervention that can be seamlessly integrated into conventional rehabilitation protocols without posing significant risks to patients. Given its passive nature, guided imagery does not require physical exertion, making it particularly suitable for individuals with severe motor impairments or those at risk of secondary complications from intensive physical training.

However, this review also has limitations that should be considered when interpreting its findings. While the comprehensive search strategy is a strength, the restriction to English-language publications introduces the possibility of language bias. Potentially valuable research published in other languages may have been excluded, which could limit the generalizability of the review’s conclusions, particularly if non-English studies report different findings or highlight unique cultural or contextual factors relevant to guided imagery interventions. Another limitation stems from the heterogeneity of the included studies. While the review focused on RCTs to maximize methodological rigor, variations in intervention protocols (e.g., type of guided imagery, duration and frequency of sessions, combination with other therapies), participant characteristics (e.g., stroke severity, time since stroke, comorbidities), and outcome measures pose a challenge for synthesizing the data and drawing definitive conclusions about the effectiveness of guided imagery. This heterogeneity makes it difficult to conduct a meta-analysis to quantitatively pool the results of the included studies. While the narrative synthesis approach adopted in the review addresses this limitation to some extent by providing a qualitative overview of the findings, it also acknowledges the inherent complexities in comparing and contrasting studies with diverse methodologies. Finally, the review acknowledges the inherent challenge of definitively linking observed improvements in motor function directly to guided imagery itself. As many studies combined guided imagery with other rehabilitation interventions, such as conventional physical therapy or occupational therapy, it can be difficult to isolate the specific contribution of guided imagery to the observed outcomes.

## 5. Conclusions

In conclusion, guided imagery, particularly MI, shows promise as a valuable adjunct in stroke rehabilitation, potentially enhancing motor recovery across various domains like gait, upper limb function, and balance. Combining MI with technologies like BCIs, robotics, and neurofeedback may further optimize outcomes by leveraging neuroplasticity mechanisms. While the evidence suggests positive trends, methodological limitations in many studies, including risk of bias and heterogeneity, necessitate cautious interpretation. Future research should prioritize rigorous, large-scale RCTs with standardized protocols and long-term follow-ups to solidify these findings and establish best practices. Ultimately, personalized guided imagery interventions, tailored to individual patient needs, hold significant potential for improving post-stroke motor rehabilitation.

## Figures and Tables

**Table 1:** Overview of guided imagery techniques and motor imagery in stroke rehabilitation.

Guided Imagery Techniques	Mechanism of Action	Key Features	Neural Correlates	Application Context	Neurophysiological Measurements	Mode of Delivery (Who Provides It)
Visual Imagery [49]	Activates visual and motor cortices, facilitating action representation.	Patients imagine observing themselves performing a movement.	Involves the occipital lobe, premotor cortex, and posterior parietal cortex.	Used for motor learning, action observation therapy, and rehabilitation planning.	EEG: brainwave patterns reflecting visual and motor cortex activity, fMRI: blood flow changes in motor-related areas.	Delivered by a therapist in structured rehabilitation sessions or through self-guided exercises with supportive instructions.
Kinesthetic Imagery [50]	Engages sensorimotor circuits via imagined proprioceptive feedback.	Patients mentally feel the sensation of movement.	Activates the M1 and somatosensory cortex.	Often integrated into sensorimotor training and rehabilitation for proprioceptive deficits.	EEG: SMR activity, TMS: cortical excitability and motor output changes, fMRI: increased activity in M1 and somatosensory cortex.	Delivered by a therapist during physiotherapy sessions, or through self-practice in conjunction with physical rehabilitation.
Guided Verbal Imagery [51]	Stimulates auditory and motor cortices through verbal instructions.	Therapist provides structured guidance, emphasizing details like force and speed.	Involves the auditory cortex, Broca’s area, and prefrontal cortex.	Frequently used in structured rehabilitation sessions and cognitive-motor interventions.	EEG: increased theta and alpha waves in auditory and motor cortices, fMRI: activation in auditory and language-related brain regions.	Delivered by a clinician or therapist who provides verbal cues and instructions, often in a clinical setting.
Technology-Assisted Imagery [52]	Enhances mental practice through VR, BCI, or robotics.	Provides real-time biofeedback and an immersive experience.	Engages the sensorimotor cortex, cerebellum, and parietal lobes.	Applied in neurorehabilitation settings to enhance engagement and monitor progress.	BCI: real-time neural feedback on motor intentions, EEG: cortical activity linked to motor imagery, fMRI: real-time monitoring of sensorimotor cortex.	Delivered through VR systems, robotic devices, or BCI systems under the supervision of a therapist or clinical technician.
Motor Imagery [53]	Simulates movement without actual execution, reinforcing motor networks.	Patients rehearse specific movements, often combined with physical training.	Activates the M1, SMA, and basal ganglia.	Integrated with conventional physiotherapy, mirror therapy, and neuromodulation.	EEG: changes in sensorimotor rhythm, TMS: assessment of motor cortical excitability, fMRI: activation in M1 and SMA during imagined movements.	Delivered by a therapist in structured rehabilitation settings, or self-guided through applications and virtual tools.

Legend: electroencephalography (EEG), functional magnetic resonance imaging (fMRI), sensorimotor rhythm (SMR), transcranial magnetic stimulation (TMS), primary motor cortex (M1), virtual reality (VR), brain–computer interface (BCI), supplementary motor area (SMA).

**Table 2:** Detailed summary of the systematic review methodology.

Section Methodology	Details
Search Strategy	**Databases:** PubMed, Web of Science, Embase, EBSCOhost, and Scopus. **Keywords/Search String:** (All Fields: “Guided Imagery”) AND (All Fields: “Stroke”) AND (All Fields: “Motor Rehabilitation”). Boolean operators and controlled vocabulary (for example, MeSH terms) were utilized.
Search Period	**Search Time Range:** no particular time frame. **Time Search Conduction:** from 15 November 2024 to 9 January 2025.
Study Selection	Two reviewers (AC, AM) independently screened articles at the title, abstract, and full-text levels. Disagreements were resolved through discussion or by a third reviewer (RSC). The PRISMA flowchart was used to visualize the selection process.
Tool Used	RoB 2 was used to assess the risk of bias in RCTs. Kappa statistic was applied to measure inter-rater agreement (threshold for substantial agreement: kappa > 0.61). Microsoft Excel allowed for efficient management of large datasets, enabling reviewers to systematically record study characteristics, risk of bias assessments, and outcome data.
Inclusion Criteria	The criteria for including studies in this systematic review aimed to find research that examined the effects of guided imagery methods, particularly motor imagery, on rehabilitation results for stroke patients. The included studies focused on adult participants (≥18 years) diagnosed with a stroke and exhibiting motor impairments. The analysis focused on guided imagery methods provided via technological tools like BCI or robotic devices, or overseen by a human facilitator, such as a clinician or therapist. The review consisted of original research that included solely RCTs.
Exclusion Criteria	Studies were eliminated if they examined interventions not related to guided imagery, including pharmacological methods, or general physical rehabilitation where guided imagery was not the primary focus. Studies focusing on populations other than stroke patients, such as those with mixed neurological conditions that did not provide a separate analysis for stroke participants, were also excluded. Articles that did not offer sufficient information on the framework, length, or execution of guided imagery protocols, or that did not employ validated clinical, neurophysiological, or functional metrics to evaluate results, were omitted. Reviews, theoretical articles, or meta-analyses were excluded, since the objective of the review was to consolidate primary experimental data. Ultimately, research published in languages other than English, or accessible solely as abstracts, posters, or conference proceedings, was eliminated because of insufficient data.
Data Extraction	Extracted data included study design, sample size, participant characteristics, intervention details (e.g., guided imagery techniques, duration), assessed outcomes (e.g., motor improvements), and findings. Data extraction and organization were facilitated using Microsoft Excel, which streamlined the process and minimized human error.
Synthesis Approach	Narrative synthesis combined with quantitative analysis. Effect sizes, evidence certainty, and statistical outcomes were analyzed to account for varied stroke conditions. A multidisciplinary team ensured a balanced interpretation of the data.
PICO Evaluation	**Population:** adult patients with a stroke diagnosis. **Intervention:** guided imagery techniques to promote motor rehabilitation. **Comparison:** traditional motor rehabilitation treatments, such as physical therapy alone, or no intervention at all. **Outcome:** gains in motor function, mobility, and overall recovery rates, while secondary goals include quality of life, patient adherence to therapy, and cognitive engagement throughout rehabilitation.

Legend: Preferred Reporting Items for Systematic Reviews and Meta-Analyses (PRISMA), Cochrane Risk of Bias 2 (RoB 2), brain–computer interface (BCI), randomized controlled trials (RCTs).

**Table 3:** Summary of the included studies.

Author	Aim	Study Design, Sample Size, and Demographic Data	Treatment Period	Intervention and Control Group	Outcomes Measures	Main Findings	Effect Size/Certainty of Evidence	Guided-Motor Imagery Intervention/Erogation of Guided Imagery
Ma et al., 2024 [55]	To investigate the effects of an MI-based BCI rehabilitation program on upper limb and hand function in patients with chronic hemiplegia following a stroke.	Study design: RCT. Size: 40 patients. Age: not specified. Sex: not specified. Diagnosis: post-stroke upper extremity hemiparesis.	Two weeks.	BCI group: 20 participants (46 initially screened, 6 withdrew). Control group: 20 participants (46 initially screened, 6 withdrew).	FMA, FMA-UE, and fMRI.	The BCI group showed significant improvement in upper extremity motor function compared to the control group. fMRI analysis revealed different patterns of brain activation and functional connectivity in the BCI group compared to the control group, particularly in motor-related areas and the default mode network.	Effect size: a correlation analysis indicated that the rise in the FMAUE score demonstrated a positive correlation with the average zALFF of the opposite precentral gyrus (r = 0.425, *p* < 0.05) and the average zReHo of the right cuneus (r = 0.399, *p* < 0.05). Certainty of evidence: moderate based on the strong study design, even if it lacked some demographic detail.	Guided/motor imagery intervention: The BCI group participated in ten 40 min MI-BCI training sessions over two weeks. These sessions were in addition to standard physical and occupational therapy, which both groups received. Erogation of guided imagery: Participants were instructed to mentally simulate grasping actions when presented with a visual cue (an arrow image). During task-state fMRI, they alternated between executing actual grasping motions or relaxation based on visual cues (closed fists or relaxed hand images) and performing MI in response to the arrow.
Pichiorri et al., 2015 [56]	To investigate the effectiveness of BCI-supported MI training compared to traditional MI training for improving motor function in stroke patients.	Study design: RCT. Size: 28 patients. Age: 18–80 years old. Sex: not specified. Diagnosis: included patients with a history of first-ever unilateral, cortical, subcortical, or mixed stroke (ischemic or hemorrhagic) 6 weeks to 6 months prior to study inclusion.	One month, with three weekly sessions.	BCI group (BCI-supported MI training): 14 patients. Control group (MI training without BCI): 14 patients.	FMA-UE, NIHSS, MRC MAS, and EEG.	Patients who received BCI-supported MI training showed greater improvements in arm function compared to those who received traditional MI training.	Effect size: Every patient gained confidence in managing the system, and no noteworthy alterations in average performance were noted from the second (66 ± 25.7%) to the final (65.1 ± 24%) BCI training session (dependent-sample t-test, *p* > 0.05). A comparative evaluation (one-tailed paired-sample t-test; *p* < 0.05 significance level) of negative t-values indicated the desynchronization patterns linked to MI tasks during the EARLY and LATE training sessions. Certainty of evidence: moderate due to the small sample size.	Guided/motor imagery intervention: The BCI group received BCI-supported MI training, which means that their brain activity was used to control a computer cursor on a screen. The control group received traditional MI training, which did not involve any BCI technology. Erogation of guided imagery: BCI. The modality of guided imagery was kinesthetic, which means that patients focused on the sensations of movement in their affected limb.
Zafar et al., 2024 [57]	To determine the additional effects of MI practice combined with dual-task training on balance and mobility in stroke patients.	Study design: RCT. Size: 30 participants. Age: mean age of 52.63 ± 8.78 years in the experimental group and 52.56 ± 7.78 years in the control group. Sex: 8 male and 7 female (experimental group); 6 male and 9 female (control group). Diagnosis: stroke.	Eight weeks.	Intervention group: n = 15. Control group: n = 15.	BBS, FRT, TUGT, MIQ-3.	The experimental group (dual-task training with MI) showed significantly greater improvements in mobility, dynamic balance, and MI ability compared to the control group (dual-task training alone) at the final assessment. While both groups improved significantly within themselves over the eight weeks on all measures, the between-group differences favored the combined intervention.	Effect size: TUGT: *p* = 0.05; BBS: *p* = 0.05; MIQ-3 (internal imagery): *p* = 0.03; MIQ-3 (external imagery): *p* = 0.037; MIQ-3 (kinesthetic): *p* = 0.004; MIQ-3 total: *p* = 0.009. Certainty of evidence: moderate due to the small sample size.	Guided/motor imagery intervention: The experimental group received dual-task training combined with MI practice. They performed seven designed tasks for four minutes each, with maximal repetitions, while simultaneously engaging in MI of the task. Erogation of guided imagery: The guided imagery was delivered through instruction by a trained physical therapist to imagine the performance of the tasks they were physically practicing. It focused on kinesthetic imagery, involving the imagined sensations of movement.
Ang et al., 2015 [58]	To compare the effectiveness of two rehabilitation interventions for chronic upper extremity impairment caused by stroke. One intervention used EEG-based MI (BCI-Manus), and the other used robotic guidance (Manus).	Study design: RCT. Size: 26 participants. Age: the average age of participants was 51.4 years. Sex: male: 16 (61.5%); female: 10 (38.5%). Diagnosis: included patients with first-ever clinical ischemic or hemorrhagic stroke within 3 h of the event, post-stroke duration > 3 months.	4 weeks, with 3 sessions per week, and each session lasting 1.5 h.	BCI-Manus group (EEG-based MI BCI with Manus robotic feedback): 11 participants. Control group (Manus group): (Manus robotically guided exercises): 15 participants.	FMMA and EEG.	Both groups showed significant improvements in FMMA scores after the intervention period. However, there was no significant difference in the improvements between the two groups.	Effect size: no significant differences were detected between the groups (*p* > 0.05). Certainty of evidence: moderate due to the small sample size.	Guided/motor imagery intervention: This intervention used a BCI system to detect MI signals from the participant. The BCI signals were then used to control a robotic arm, which provided feedback to the participant. Erogation of guided imagery: the MI was guided by the participant’s imagination of moving their affected arm. The BCI system detected these imagined movements and provided feedback through the robotic arm.
Ang et al., 2015 [59]	To assess the effects of tDCS on the rehabilitation of upper limbs following stroke, specifically in conjunction with EEG-based MI and robotic feedback.	Study design: RCT. Size: 19 patients. Age: the average age of participants was 54.1 years old, with a range of 42.1 to 68.4 years. Sex: fourteen participants were male (73.7%) and five were female (26.3%). Diagnosis: first-ever subcortical stroke at least 9 months before recruitment.	Two weeks, with rehabilitation sessions occurring five times per week for a total of ten sessions.	tDCS group: 10 patients. Control group (sham group): 9 patients.	FMMA, EEG, and online accuracy of MI detection and the ERD laterality coefficient were evaluated during the rehabilitation sessions.	No significant improvements in FMMA scores were observed at the two-week mark in either the tDCS or sham group. However, at the two-week follow-up, both groups showed significant improvements in FMMA scores compared to baseline. The online accuracy of MI detection was significantly higher in the tDCS group compared to the sham group across the rehabilitation sessions.	Effect size: The *p*-value for the difference in FMMA scores between the tDCS and sham groups at week 2 was not significant (*p* > 0.36). The *p*-value for the difference in FMMA scores between the tDCS and sham groups at week 4 was not significant (*p* > 0.874). The *p*-value for the difference in online accuracy of MI detection between the tDCS and sham groups was significant (*p* = 0.002). Certainty of evidence: moderate due to the small sample size and the lack of significant differences in motor improvements between the tDCS and sham groups.	Guided/motor imagery intervention: the intervention involved guided MI, where participants imagined moving their stroke-affected upper limb while receiving visual cues. Erogation of guided imagery: the guided MI was delivered through visual instructions displayed on a screen.
Ang et al., 2010 [60]	To investigate the ability of stroke patients to use EEG-based MI-BCI and the effectiveness of this intervention for improving motor function in the upper limbs.	Study design: RCT. Size: 25 patients (26 initially randomized, 1 dropped out). Age: MI-BCI group: 47.5 ± 13.5 years old; robotic rehabilitation group: 53.1 ± 8.6 years old. Sex: MI-BCI group: 9 male (81.8%), 2 female (18.2%). Robotic rehabilitation group: 7 male (50%), 7 female (50%). Diagnosis: Included hemiparetic stroke patients.	4 weeks—12 sessions, 1 h each.	MI-BCI with robotic feedback group: 11 patients. Control group (robotic rehabilitation group): 14 patients	FMA.	Both groups showed significant improvements in FMA scores at post-rehabilitation and 2-months follow-up. The robotic rehabilitation group showed greater improvements at week 2 compared to the MI-BCI group.	Effect size: At the two-month follow-up, both groups maintained statistically significant improvements from baseline. The MI-BCI group had a *p*-value of 0.020, while the robotic rehabilitation group’s was 0.013. Certainty of evidence: moderate, since the study provides evidence that EEG-based MI-BCI with robotic feedback can be used by stroke patients and may be effective for motor improvement	Guided/motor imagery intervention: patients were instructed to perform MI of the affected upper limb based on visual cues. Erogation of guided imagery: visual cues were used to instruct patients on when to perform MI.
Mihara et al., 2013 [61]	To investigate the efficacy of NIRS-mediated neurofeedback combined with MI training for improving upper limb motor function in patients with chronic stroke.	Study design: RCT. Size: 20 patients. Age: 40–68 years. Sex: 12 males and 8 females. Diagnosis: included patients with first-time subcortical stroke, motor hemiparesis, no sensory loss, no cognitive disturbance, no depression, no carotid stenosis, no history of neurological disorders, age ≥ 20 years, and ≥12 weeks from initial symptom onset.	Two weeks, with participants receiving mental practice with MI accompanied by neurofeedback three times a week.	REAL feedback group: 10 patients. Control group (SHAM feedback group): 10 patients	FMA, ARAT, MAL, KVIQ-10.	Patients in the REAL group showed significant improvements in the hand/finger subscale of the FMA compared to the SHAM group. There was no significant difference between the groups in terms of the total FMA score, the ARAT, or the MAL. The amount and quality of paretic arm use assessed by MAL were improved in both groups. There was no significant change in subjective skills in kinesthetic or visual MI in either group.	Effect size: significant improvement of hand function was observed in the REAL group compared to the SHAM group (F1,18 = 59.7; *p* < 0.001 for POST1 vs. PRE and F1,18 = 68.6; *p* < 0.001 for POST2 vs. PRE). Certainty of evidence: moderate, since it is a pilot study with a small sample size.	Guided/motor imagery intervention: the intervention consisted of mental practice with MI for 10 min, followed by kinesthetic MI training with neurofeedback for another 10 min, in each session. Erogation of guided imagery: the guided MI involved video and kinesthetic components.
Liu et al., 2023 [62]	To investigate the effect of MI-BCI training combined with conventional rehabilitation on upper limb motor function and attention in stroke patients.	Study design: RCT. Size: 60 participants. Age: 18–65 years. Sex: BCI group: 22 males and 8 females; control group: 19 males and 11 females. Diagnosis: included patients with first-onset stroke, within one month of disease duration, dominant right hand with right-sided hemiplegia, etc.	3 weeks, 5 days a week.	BCI group (MI-BCI training plus conventional rehabilitation): 30 patients. Control group (conventional rehabilitation only): 30 patients.	FMA-UE, ANT, WMFT, MBI, SDMT, and Schulte Grid Test.	MI-BCI training combined with conventional rehabilitation significantly improved upper limb motor function compared to conventional rehabilitation alone. MI-BCI training also significantly improved attention compared to conventional rehabilitation alone. Improvements in motor function were significantly correlated with improvements in attention.	Effect size: change in FMA-UE between groups: *p* < 0.001; difference in FMA-UE at final assessment: *p* = 0.049; WMFT: change in WMFT between groups: *p* < 0.001; difference in WMFT at final assessment: *p* = 0.033; change in MBI between groups: *p* < 0.001; difference in MBI at final assessment: *p* < 0.001. Certainty of evidence: high, given the large sample size and study design.	Guided/motor imagery intervention: MI-BCI training using the LSR-AII BCI rehabilitation training instrument. Patients imagined specific movements (shoulder abduction, etc.) while the BCI system recorded and analyzed EEG signals. Erogation of guided imagery: The guided imagery was delivered through computerized voice commands, on-screen animation, and real-time feedback based on the patient’s brain activity. The BCI system provided the link between the imagined movement and the FES, creating a closed-loop feedback system.
Brunner et al., 2024 [63]	To compare the effects of BCI training to standard upper limb rehabilitation in patients with severe paresis after stroke.	Study design: RCT. Size: 40 patients initially; 35 completed the 3-month follow-up. Age: BCI group: mean 56 years; control group: mean 57 years. Sex: BCI group: 11 male (57.9%), 8 female (42.1%); control group: 14 male (66.7%), 7 female (33.3%). Diagnosis: included adults with first-ever or former stroke), without upper limb motor residuals, within 60 days post-stroke, severe paresis/paralysis, able to give informed consent.	3–4 weeks, 3–4 times a week (12 sessions targeted).	BCI group: 19 patients. Control group: 21 patients.	ARAT, FMA-UL, EEG measures.	While both groups showed improvement, there was no statistically significant difference in ARAT or FMA improvement between the BCI group and the control group at 3 months. MEP status was a significant predictor of improvement, with MEP+ patients having much higher odds of achieving a clinically meaningful improvement.	Effect size: ARAT improvement within groups: *p* = 0.012 (control), *p* = 0.007 (BCI); ARAT improvement between groups: *p* = 0.328; FMA improvement between groups: *p* = 0.406; logistic regression (MEP status): *p* = 0.007. Certainty of evidence: This was a pilot study, so the certainty of evidence is considered lower compared to larger, more definitive trials. The study also had a relatively small sample size and some attrition.	Guided/motor imagery intervention: The BCI intervention used the RecoveriX system. Patients imagined opening their hand (finger and wrist extension) while wearing an EEG cap. The BCI system detected the MI, and if the classification confidence was above 50%, FES was triggered, inducing the imagined movement. Visual feedback was provided via an avatar on a screen. Erogation of guided imagery: the guided imagery was delivered through the combination of auditory cues (instructions to imagine movement), visual feedback (avatar hands on the screen), and the somatosensory feedback of the FES-induced movement.
Kashoo et al., 2022 [64]	To investigate the efficacy of anodal tDCS combined with VMI and upper limb functional training compared to VMI and upper limb functional training alone in improving upper limb function in chronic stroke patients.	Study design: RCT. Size: 64 participants. Age: experimental group: mean 58.7 years; control group: mean 59.9 years. Sex: experimental group: 25 males and 7 females; control group: 24 males and 8 females. Diagnosis: included participants with chronic stroke (stroke in the past 6 months), able to perform visual MI.	2 weeks (10 treatment sessions).	Experimental group (MI and tDCS): 32 participants. Control group (MI only): 32 participants.	FMA, ARAT.	The experimental group (tDCS + VMI + functional training) showed significant improvements in both FMA and ARAT scores from baseline. The control group (VMI + functional training) also showed significant improvements in both FMA and ARAT scores from baseline.	Effect size: between-group difference in FMA: *p* < 0.001; η² = 0.37; between-group difference in ARAT: *p* < 0.001; η² = 0.38. Certainty of evidence: The study acknowledges limitations, including the small sample size, the potential for bias due to lack of blinding effectiveness evaluation, and the inability to isolate the individual contributions of MI and functional training. Therefore, the certainty of the evidence is moderate.	Guided/motor imagery intervention: Participants in the experimental group underwent VMI. They watched video and audio tapes of upper limb activities, then performed mental imagery of the movements while receiving tDCS. This was followed by actual performance of the activities. Erogation of guided imagery: the guided imagery was delivered through video and audio tapes demonstrating the activities, combined with concurrent tDCS and subsequent physical practice of the movements.
Mihara et al., 2021 [65]	To investigate the feasibility and efficacy of fNIRS-NFB combined with gait and balance-related MI for improving gait disturbance in patients with subcortical stroke.	Study design: RCT. Size: 57 patients were initially enrolled, but 3 declined before intervention. Age: real feedback group: mean 62.25 years; sham feedback group: mean 60.08 years. Sex: real feedback group: 21 male, 7 female; sham feedback group: 19 male, 7 female. Diagnosis: real feedback group: mean 62.25 years; sham feedback group: mean 60.08 years.	2 weeks, 3 sessions per week (6 sessions total).	Real feedback group: 28 patients. Control group: sham feedback group: 26 patients.	FIM, F-M, BBS, and gait speed.	The real feedback group showed significantly greater improvement in TUG time compared to the sham feedback group. The real feedback group also demonstrated greater improvement in the BBS score. fNIRS data showed increased SMA activation during walking imagery in the real feedback group, and resting-state functional MRI revealed increased connectivity between the SMA and specific brain regions.	Effect size: TUG improvement between groups: *p* = 0.028 (absolute difference), *p* = 0.042 (recovery rate); BBS improvement between groups: *p* < 0.001; interaction between time and intervention (TUG): *p* = 0.030; interaction between time and intervention (BBS): *p* < 0.001. Certainty of evidence: while the study design is robust (double-blind, randomized), it is considered exploratory, so the certainty of evidence is moderate.	Guided/motor imagery intervention: Patients in the real feedback group performed kinesthetic MI of “rising up from the chair and stepping twice” and “walking along the corridor” tasks. They received real-time visual feedback of their SMA activation during these imagery tasks. Erogation of guided imagery: the guided imagery was facilitated by a 10 min video instruction demonstrating the tasks, followed by real-time visual feedback of SMA activation during the imagery practice.
Ang et al., 2012 [66]	To investigate the effects of tDCS combined with EEG-based MI-BCI and robotic feedback on upper limb stroke rehabilitation, compared to sham-tDCS.	Study design: RCT. Size: 19 patients. Age: not specified. Sex: not specified. Diagnosis: hemiparetic stroke patients.	2 weeks, 5 times a week (10 rehabilitation sessions).	tDCS group: 3 patients. Control group (sham-tDCS group): 2 patients.	Online accuracy of detecting MI during evaluation sessions. Offline accuracy of classifying MI during therapy sessions.	In total, 68% of screened patients operated the MI-BCI better than chance level. Average online accuracy across rehabilitation sessions was approximately 67% for both tDCS and sham-tDCS groups, with no clear difference between groups. Offline analysis suggested that averaged accuracies of the tDCS group across rehabilitation sessions were higher than the sham-tDCS group, but this difference was not statistically significant due to small sample size and variability.	Effect size: statistical significance between tDCS and sham-tDCS groups for online and offline accuracies was not reached due to the small sample size. Certainty of evidence: the certainty of evidence is low due the small sample size and the findings are preliminary.	Guided/motor imagery intervention: Patients performed MI of the stroke-affected upper limb. During therapy sessions, if MI was detected, the MIT-Manus robot provided movement feedback, moving the affected limb towards a target on the screen. Erogation of guided imagery: the guided imagery was performed with visual cues and robotic feedback contingent on the detection of MI via the BCI.
Kang et al., 2021 [67]	To investigate the effects of adding ES to LF-rTMS and audio-based MI on upper extremity motor function in subacute stroke patients.	Study design: RCT. Size: 20 participants. Age: 20 years or older. Sex: rTMS + MI + ES group: 6 males and 2 females; rTMS + MI: 4 males and 5 females. Diagnosis: included patients with first-ever stroke, 1 week to 3 months post-stroke onset, no previous upper extremity functional impairments.	2 weeks, 5 days a week (10 sessions).	Experimental group (rTMS + audio-based MI + active ES): 8 participants; control group (rTMS + audio-based MI + sham ES): 9 participants.	FMA-UE, hemiplegic shoulder abduction and finger extension score (modified Medical Research Council scale), MBI, Purdue Pegboard Test, and FFT.	Both groups showed improvement in FMA scores, but the improvement was significantly greater in the experimental group (rTMS + audio-based MI + active ES) compared to the control group (rTMS + audio-based MI + sham ES). The experimental group also showed significant improvement in shoulder abduction and finger extension scores.	Effect size: FMA-UE change between groups: *p* = 0.04; shoulder/elbow/forearm FMA subscore change between groups: *p* = 0.03. Certainty of evidence: The study has a small sample size and is from a single center, which limits the generalizability of the findings. Therefore, the certainty of evidence is moderate.	Guided/motor imagery intervention: Participants followed 20 min audio-recorded instructions to imagine UE motions and daily movements. The MI training was divided into four parts: imagination preparation, MI warm-up, imagining activities of daily living, and cool-down. Erogation of guided imagery: The guided imagery was delivered via audio recordings, with an occupational therapist explaining the content beforehand to ensure understanding.
Kim et al., 2025 [68]	To compare the effects of MI-contingent feedback BCI and MI-independent feedback BCI on upper limb function in chronic stroke patients.	Study design: RCT. Age: MI-contingent feedback BCI group: mean 49.0 years. MI-independent feedback BCI group: mean 46.0 years. Sex: MI-contingent feedback BCI group: 10 male, 2 female. MI-independent feedback BCI group: 9 male, 4 female. Diagnosis: included individuals with hemiplegia due to a first-ever stroke with unilateral hemisphere lesions, in the chronic stage of stroke (≥6 months post-onset), with affected wrist extensor muscle weakness.	4 weeks, 5 days a week (20 sessions).	MI-contingent feedback BCI group: 12 participants (initially 14, but 2 dropped out); control group (MI-independent feedback BCI group): 13 participants.	Changes in MRC-WE and active range of motion in wrist extension at 4 weeks. FMA, BBT, SIS, and resting-state EEG measurements.	The MI-contingent feedback BCI group showed significantly greater improvement in MRC-WE compared to the MI-independent feedback BCI group at 4 weeks. AROM-WE also improved significantly in the MI-contingent feedback BCI group but not in the MI-independent feedback BCI group. Resting-state EEG showed significant time × group interactions for effective connectivity in the ipsilesional premotor area (β frequency band) and improvements in functional connectivity in the MI-contingent feedback BCI group.	Effect size: Difference in MRC-WE change between groups: *p* = 0.036. Significant improvements within the MI-contingent feedback BCI group for MRC-WE (*p* = 0.002) and AROM-WE (*p* = 0.019) at W4 compared to W0. Significant time × group interaction in PDC-based functional connectivity was observed in the ipsilesional premotor area (*p* = 0.005 and *p* = 0.014). Certainty of evidence: Moderate. The study is an RCT with blinding, which strengthens the evidence. However, the sample size is relatively small, and the study focuses on chronic stroke patients with specific inclusion criteria, which may limit generalizability.	Guided/motor imagery intervention: participants were instructed to imagine wrist dorsiflexion according to visual and verbal cues, alternating between “left” and “right” hands in a semi-random order. Erogation of guided imagery: the guided imagery was delivered through visual cues (arrows on a monitor), verbal instructions, and contingent feedback (FES and virtual avatar movement) in the MI-contingent group or non-contingent feedback (FES) in the MI-independent group.
Luo, 2024 [69]	To investigate the effect of combining MI-based, BCI-controlled electrical stimulation with conventional rehabilitation on lower limb motor function, walking ability, and activities of daily living in patients with acute ischemic stroke.	Study design: RCT. Size: 64 patients. Age: experimental group: ≤40: 2 patients (6.3%); 41–60: 19 patients (59.4%); 60: 11 patients (34.4%). Control group: ≤40: 1 patient (3.1%); 41–60: 15 patients (46.9%); 60: 16 patients (50%). Sex: experimental group: 23 male, 9 female; control group: 19 male, 13 female. Diagnosis: Stroke. Included patients with a disease duration of 2 weeks or less, first diagnosed with acute cerebral infarction, unilateral hemiparesis, and motor dysfunction of the affected lower limb.	2 weeks, 20 sessions (1 h per day, 30 min in the morning and 30 min in the afternoon).	Experimental group (BCI rehabilitation + conventional rehabilitation): 32 patients. Control group (conventional rehabilitation): 32 patients.	FMA-LE, FAC, and MBI.	The experimental group (BCI rehabilitation + conventional rehabilitation) showed significantly greater improvement in FMA-LE scores compared to the control group (conventional rehabilitation only) after the 2-week intervention. The experimental group also showed statistically significant improvements in FAC and MBI scores compared to the control group.	Effect size: FMA-LE score difference between groups post-intervention: *p* < 0.001; FAC score difference between groups post-intervention: *p* = 0.031; MBI score difference between groups post-intervention: *p* < 0.001. Certainty of evidence: Moderate. The study is an RCT, which strengthens the evidence. However, it is a single-center study, and the relatively short intervention period and lack of long-term follow-up may limit the generalizability of the findings.	Guided/motor imagery intervention: patients performed MI of foot dorsiflexion, foot inversion, knee extension, and calf external rotation, guided by voice prompts and a VR screen. Erogation of guided imagery: the guided imagery was delivered through voice prompts, a VR screen displaying movements, and contingent FES triggered by the BCI system when MI reached a certain threshold.
Frolov et al., 2017 [70]	To investigate the effectiveness of BCI-controlled hand exoskeleton training combined with standard physical therapy for upper extremity motor function recovery in stroke patients.	Study design: RCT. Size: 74 patients. Age: The median age was 58 (50.0; 65.0) years for the BCI group and 58.0 (52.0; 67.0) years for the control group. Sex: 61.8% (34) of the BCI group were male and 73.7% (14) of the control group were male. Diagnosis: included patients with subacute (1–6 months from onset) or chronic (>6 months from onset) stroke; hand paresis, mild to plegia; a single focus of ischemic or hemorrhagic stroke with a supratentorial localization.	10 daily sessions, each lasting 30 min, conducted every day with breaks on weekends and holidays (up to 3 consecutive days).	BCI group: 55 patients. Control group: 19 patients.	FMMA, ARAT, MAS.	Both groups showed improvement, but the BCI group exhibited significantly greater improvements in ARAT scores, particularly in grasp, pinch, and gross movements. FMMA scores also improved significantly in both groups, but the improvement trend favored the BCI group.	Effect size: Significant improvements (*p* < 0.01) in ARAT (grasp, pinch, gross movement) and FMMA scores within the BCI group. Significant difference (*p* < 0.05) between BCI and control groups in ARAT total score improvement. Certainty of evidence: The study reports some adverse events (headache, fatigue), although none led to withdrawal. Therefore, the certainty of evidence is moderate.	Guided/motor imagery intervention: patients were instructed to perform MI of left-hand opening, right-hand opening, or relaxation, guided by visual cues on a computer screen. Erogation of guided imagery: The guided imagery was delivered through visual cues (colored arrows) on a computer screen. Feedback was provided online, both visually (marker color change) and kinesthetically (exoskeleton movement).
Pan et al., 2019 [71]	To investigate whether the combination of 1 Hz rTMS and audio-based MI training is more effective than rTMS alone for improving upper limb motor function in chronic stroke patients.	Study design: RCT. Size: 42 patients. Age: rTMS + MI group: mean 63.38 years; rTMS group: mean 64.14 years. Sex: rTMS + MI group: 16 male (76%); rTMS group: 12 male (57%). Diagnosis: included patients diagnosed with ischemic stroke for the first time; inpatients within 3 to 12 months from stroke onset; without prior upper limb function impairments.	2 weeks, 10 sessions (5 days/week), 30 min per session for both rTMS and MI/relaxation. Patients also received 120 min of conventional rehabilitation daily.	rTMS + MI group (rTMS with MI): 21 patients. Control group: rTMS group (rTMS with audiotape-led relaxation): 21 patients.	WMFT, FMA-UE, MBI, BBT.	Both groups showed improvement in all outcome measures after the 2-week intervention. The rTMS + MI group demonstrated significantly greater improvements in WMFT, UE-FMA, MBI, and BBT scores compared to the rTMS-only group. These findings suggest that combining rTMS with MI training is more effective than rTMS alone for upper limb rehabilitation after stroke.	Effect size: Significant differences (*p* < 0.01) between groups at weeks 2 and 4 for WMFT, UE-FMA, and BBT. Significant difference (*p* < 0.001) between groups at weeks 2 and 4 for MBI. Certainty of evidence: Moderate. The study uses an RCT design, which is a strong point. However, it is a single-center trial, and the lack of a conventional rehabilitation-only control group is a limitation. The sample size is relatively small.	Guided/motor imagery intervention: participants imagined everyday activities (writing, opening doors, drinking, etc.) for 30 min, guided by audio instructions, including an initial 3 min relaxation period, 10 min of warm-up exercises, 15 min of ADL practice, and a 2 min cool-down. Erogation of guided imagery: The guided imagery was delivered via audio instructions. Soothing music was included in the background of the audio in the relaxation group.
Oostra et al., 2015 [72]	To investigate the effect of a 6-week MIT program on MI ability and gait performance in chronic stroke patients, compared to an MR control group.	Study design: RCT. Size: 44 patients. Age: MIT group: mean 50.3 years; MR group: mean 53.7 years; control group: mean 47.3 years. Sex: MIT group: 15 male, 6 female; MR group: 14 male, 9 female; control group: 14 male, 13 female. Diagnosis: included patients with a first-ever stroke less than one year prior; able to walk 10 m with minimal assistance.	6 weeks of training, with 30 min daily sessions for the MIT and MR groups, in addition to standard rehabilitation.	MIT group: 21 patients; MR group: 23 patients; healthy control group: 27 subjects.	MIQ-RS, Walking Trajectory Test, 10 m walk test (gait velocity), FMA-LE.	Both groups showed improvement in gait velocity and FMA-LE scores after 6 weeks. The MIT group demonstrated significantly greater improvement in kinesthetic MI ability (MIQ-RSkin) compared to the MR group. The MIT group also showed a significantly greater reduction in walking duration (improved gait velocity) compared to the MR group.	Effect size: Significant within-group improvements (*p* < 0.001) in gait velocity and FMA-LE scores for both groups. Significant interaction effect (*p* < 0.05) between treatment type and assessment time for MIQ-RSkin, favoring the MIT group. Significant interaction effect (*p* < 0.05) for the 10 m walk test, favoring the MIT group. See Table III for more specific *p*-values. Certainty of evidence: Moderate. The study design includes randomization and a control group, which are strengths. However, it is a single-center trial, and while the assessor was blinded, the therapists delivering the interventions were not.	Guided/motor imagery intervention: participants in the MIT group received individual 30 min sessions, starting with relaxation, then progressing through imagery of everyday movements, gait-specific movements (based on individual gait analysis), and finally, gait exercises embedded in ADL. Erogation of guided imagery: The guided imagery was delivered through verbal instructions from the therapist, focusing on internal visual and kinesthetic perspectives. Auditory cues were used to guide walking speed imagery.
Kiloatar et al., 2024 [73]	To objectively evaluate changes in the ANS during AO, MI, and AE interventions in patients with chronic stroke using HRV.	Study design: RCT. Size: 36 participants. Age: MI group: mean 58.50 years; AO group: mean 62.73 years; AE group: mean 65.09 years. Sex: not specified. Diagnosis: at least 6 months post-stroke, able to walk at least 45 m (with or without support).	The interventions (MI, AO, and AE) were each a single 5 min session. The study design focused on the immediate ANS response to these interventions rather than a longer treatment period.	MI group: 10 participants; AO group: 15 participants; AE group: 11 participants.	Blood pressure (systolic and diastolic), heart rate, HRV parameters.	In the AE group, HR significantly increased, and the mean RR interval significantly decreased after walking. In the AO group, lnRMSSD significantly decreased after watching the walking video. Heart rate was significantly higher in the AE group compared to the other two groups.	Effect size: significant difference (*p* < 0.05) within the AO group for lnRMSSD before and after intervention. Certainty of evidence: Moderate. The study uses an RCT design, which is a strength. However, it is a single-center study with a relatively small sample size. The interventions were also very brief (5 min).	Guided/motor imagery intervention: Participants were given a short training session on how to perform kinesthetic imagery, focusing on proprioceptive and kinesthetic sensations of walking. They then performed 5 min of MI while HR and HRV were recorded. A pre-prepared scenario was used to verbally guide the imagery. Erogation of guided imagery: the guided imagery was delivered verbally by the physiotherapist, focusing on kinesthetic sensations.
Cho et al., 2013 [74]	To examine the effects of MI training combined with gait training on the balance and gait abilities of chronic stroke patients.	Study design: RCT. Size: 28 participants. Age: experimental group: mean 53.93 years; control group: mean 53.85 years; Sex: experimental group: 9 male, 6 female; control group: 8 male, 5 female. Diagnosis: more than six months after stroke onset, no auditory or visual problems, ability to walk > 10 m independently, not taking medication that could influence balance or gait.	6 weeks, MI training (15 min) followed by gait training (30 min) three times a week.	Experimental group (MIT with gait training): 15 patients; control group (gait training only): 13 patients.	FRT, TUGT, 10 m walk test, FMA.	Both groups showed significant improvements in FRT, TUGT, and 10 m walk test after the intervention. The experimental group showed significant improvement in the FMA, while the control group did not.	Effect size: FRT: *p*-value = 0.046; TUG: *p*-value = 0.038; 10 m walk test: *p*-value = 0.035; FMA-LE: *p*-value = 0.029. Certainty of evidence: Moderate. The study design includes randomization and blinding of participants, researchers, and research assistants involved in the program and measurements, which are strengths. However, the small sample size is a limitation that affects the certainty of the evidence.	Guided/motor imagery intervention: Participants in the experimental group underwent 15 min of MIT prior to their 30 min gait training sessions. The training involved watching videos of normal gait, explanations of normal gait by a researcher, and imagining normal gait movements based on the visual materials. Subjects were asked to explain the movement they were imagining. Erogation of guided imagery: The guided imagery was delivered using visual materials (videos of normal gait) and verbal explanations by the researcher. The subjects were then prompted to verbally explain the movement they were imagining.
Ietswaart et al., 2011 [75]	To evaluate the therapeutic benefit of mental practice with MI in sub-acute stroke patients with moderate motor weakness.	Study design: RCT. Size: 121 participants. Age: MIT group: mean 69.3 years; attention-placebo control group: mean 68.6 years; normal care control group: mean 64.4 years. Sex: MIT group: 23 male, 18 female; attention-placebo control group: 22 male, 17 female; normal care control group: 25 male, 16 female. Diagnosis: history of stroke 1–6 months prior	4 weeks, with 45 min training sessions 3 days a week and 30 min independent sessions twice a week.	MIT group: 41 patients; attention-placebo control group: 39 patients; normal care control group: 41 patients.	ARAT, HADS, MSQ. Grip strength (dynamometer), hand function (timed manual dexterity), ADL (Barthel Index), disability (modified functional limitation profile).	No significant differences were found between the three groups on the primary outcome measure (ARAT) or any of the secondary outcome measures. Recovery was evident in all groups across all outcome variables. MI ability was not impaired as a group and correlated with improvement on motor tasks, but not with the benefit of MI practice itself.	Effect size: The main effect of time (recovery between baseline and outcome) was significant (*p* < 0.001) for all outcome variables. However, the between-group differences on all outcome measures were not significant. The effect sizes for the between-group comparisons were very small (0.001 to 0.019). Certainty of evidence: Moderate. The study design includes randomization, a detailed protocol, and blinding of assessors, which are strengths. The sample size is also relatively large.	Guided/motor imagery intervention: The MIT group received 12 supervised 45 min sessions and 8 independent 30 min sessions. The sessions included verbal facilitation, use of objects, pictorial scenes, action observation, and mental rotation of hands. The supervised sessions covered elementary movements, goal-directed movements, and ADLs. Erogation of guided imagery: The guided imagery used verbal facilitation by the therapist, visual aids (objects, pictures, mirrors, videos), and auditory guidance (taped instructions for independent practice).
Schuster et al., 2012 [76]	To investigate the effects of embedded and added MIT on the ability to perform a complex motor task (“Going down, laying on the floor, and getting up again”) in patients after stroke, and to explore the feasibility of a larger trial.	Study design: RCT. Size: 41 participants. Age: Experimental group 1: mean 65.8 years; Experimental group 2: mean 59.7 years; control group: mean 64.4 years; Sex: Experimental group 1: 3 female; 10 male; Experimental group 2: 5 female; 9 male; control group: 4 female; 9 male. Diagnosis: first ischemic or hemorrhagic stroke at least 3 months prior, able to stand for 30 s, able to walk 20 m.	Two weeks, with six physiotherapy sessions (25–30 min each) plus MI training (embedded or added).	Experimental Group 1 (embedded MI): 13 patients. Experimental Group 2 (added MI): 14 patients. Control group: 13 patients.	KVIQ, BBS. Time difference (seconds) to perform the motor task from pre- to post-intervention. Activities-Specific Balance Confidence Scale.	All three groups improved in the time taken to perform the motor task after the intervention, and this improvement remained at the two-week follow-up. There was no significant difference between the three groups in the time taken to perform the motor task. All groups also showed improvement in the help needed to perform the task.	Effect size: Significant effect of time for the motor task (F(2, 36) = 19.14, *p* < 0.001, η² = 0.35). Significant improvement for all groups in help needed (F(2, 36) = 77.37, *p* < 0.001, η² = 0.68). No significant effect for group on the motor task (F(2, 36) = 1.55, *p* = 0.199, η² = 0.079). Certainty of evidence: Moderate. This is a pilot RCT with a small sample size, which limits the generalizability of the findings. While there was improvement across groups, the lack of significant between-group differences suggests that neither embedded nor added MI provided additional benefit over standard physiotherapy.	Guided/motor imagery intervention: Embedded MI (group 1): MI was integrated into the physiotherapy sessions, with patients imagining each stage of the motor task before physically practicing it. Added MI (group 2): MI was performed after the physiotherapy session, with patients listening to a tape describing each stage of the motor task and imagining the complete task. Erogation of guided imagery: Both embedded and added MI used a combination of verbal instruction (live from the therapist or pre-recorded on tape) and internal imagery (patients imagining the movement). The embedded group also incorporated the physical practice of the movement stages.
Dickstein et al., 2013 [77]	To examine the effects of IMI practice on indoor and community ambulation, as well as fall-related self-efficacy, in community-dwelling individuals with post-stroke hemiparesis.	Study design: RCT. Size: 23 participants. Age: 60–80 years. Sex: integrated imagery group (n = 12): 3 women, 9 men; control group (n = 11): 4 women, 7 men Diagnosis: unilateral stroke 6 months to 2 years prior, limited indoor/outdoor ambulation due to stroke.	Two 4-week phases, with 15 min sessions 3 times a week.	Integrated imagery group: 13 participants (initially), 12 in final analysis. Control group: 12 participants (initially), 11 in final analysis. Integrated imagery group (crossover): 11 participants.	10 m walk test, FESS, SAM.	Gait speed significantly improved after the IMI intervention, but not after the control treatment. No significant changes were observed in community ambulation (number of steps, maximal activity). Fall-related self-efficacy showed a significant increase after IMI in one of the groups but not in the other. The difference between the groups was not significant.	Effect size: Significant interaction effect between time and intervention for gait speed (F(1,20) = 2.87, *p* < 0.1). Significant positive effect of IMI on walking speed (t11 = 3.95, *p* < 0.002). No significant effect of the control treatment on walking speed (t10 = 0.41, *p* = 0.69). Significant effect of IMI on gait speed across all subjects (F(1,21) = 11.23, *p* < 0.003). Effect size for IMI on gait speed was large and significant (ES = 0.71, *p* < 0.002). Certainty of evidence: Moderate. The small sample size and short intervention period limit the generalizability of the findings. While gait speed improved, there was a lack of significant improvement in community ambulation.	Guided/motor imagery intervention: Participants used imagery scripts based on their individual goals, imagining walking in familiar environments (home, community interior, community exterior). Sessions included relaxation exercises and both kinesthetic and visual imagery, along with motivational imagery. Erogation of guided imagery: The guided imagery used a combination of verbal instruction (from the therapist), internal imagery (patients imagining the walking scenarios), and a focus on both kinesthetic and visual sensations. Motivational imagery was also incorporated.
Verma et al., 2011 [78]	To investigate the effectiveness of TOCCT combined with MI on gait abilities in subacute stroke patients compared to conventional rehabilitation.	Study design: RCT. Size: 30 inpatients. Age: mean age was 54.16 ± 7.63 years. Sex: 22 men, 8 women. Diagnosis: first ischemic or hemorrhagic stroke at least 3 months before; able to stand for 30 s; able to walk 20 m.	Two weeks, 7 days a week.	Experimental Group 1 (embedded MI): 13 patients. Experimental Group 2 (added MI): 14 patients. Control group: 14 patients.	FAC, RVGA, 10 m walk test, comfortable and maximal speed; 6 min walk test. Barthel Index.	TOCCT combined with MI showed greater improvement in FAC, RVGA, cadence, comfortable walking speed, and 6 MWT compared to conventional rehabilitation. The experimental group also reached independent functional ambulation (FAC level 5) earlier than the control group. No significant differences were found between the groups for gait asymmetry (step length, stride length) or maximal walking speed at all time points.	Effect size: Significant differences between groups at post-intervention and follow-up for FAC, RVGA, cadence, comfortable walking speed, and 6 MWT (F: *p* = 0.001–0.049; U: *p* = 0.001). Kaplan-Meier analysis showed a significant difference in achieving FAC level 5 between groups (Mantel-Cox: *p* < 0.036). Certainty of evidence: moderate since this is a small, single-center study with a relatively short intervention period.	Guided/motor imagery intervention: The experimental group received 15 min of MI followed by 25 min of TOCCT. MI involved imagining walking abilities and tasks related to real-life situations. Participants were familiarized with MI in a pre-intervention session and asked to keep a diary of their MI practice. Erogation of guided imagery: Verbal guidance from the therapist. It can be inferred that it likely involved internal imagery (patients imagining themselves performing the actions) and likely incorporated both visual and kinesthetic components, given the nature of the imagined tasks (walking, stair climbing, etc.).
Kim et al., 2013 [79]	To investigate the effects of AO training and MI training on dynamic balance and gait ability in chronic stroke patients.	Study design: RCT. Size: 27 participants. Age: AO training group: mean 55.3 years; MIT group: mean 54.8 years; physical training group: mean 59.8 years. Sex: AO training group: 7 male, 2 female; MIT group: 6 male, 3 female; physical training group: 7 male, 2 female. Diagnosis: first-time ischemic or hemorrhagic stroke; over six months since onset; able to walk independently more than 10 m.	Four weeks, 5 times per week.	AO training group: 9 participants; MIT group: 9 participants; physical training group: 9 participants.	TUG, FRT, WAQ, FAC, spatiotemporal gait parameters (gait speed, cadence, step length, stride length, single/double limb support).	Both AO training and MIT showed improvements in dynamic balance and gait-related indicators. The AO training group showed significantly greater improvement than the physical training group in the TUG, gait speed, cadence, and single limb support on the affected side. No significant differences were found between the AO and MI groups.	Effect size: Significant difference between pre- and post-test TUG scores among the three groups (*p* < 0.05). Post hoc test revealed significant difference between AO and physical training groups (*p* < 0.05). Significant difference in gait speed among the three groups (*p* < 0.05). Post hoc test revealed significant difference between AO and physical training groups (*p* < 0.05). Certainty of evidence: moderate, since this was a small pilot study, which limits the generalizability of the findings.	Guided/motor imagery intervention: Participants in the MI group listened to a 20 min audio program guiding them through MI exercises identical to those in the AO group (pelvic tilting, trunk movements, sit-to-stand, weight shifting, gait). This was followed by 10 min of physical training based on the same content. Erogation of guided imagery: this involved both visual and kinesthetic components, given the nature of the exercises (movements, balance, gait).
Yin et al., 2022 [80]	To investigate the effects of MIT combined with conventional therapy on lower limb motor rehabilitation in stroke patients.	Study design: RCT. Size: 32 participants. Age: experimental group: mean 56.9 years. Control group: mean 57.1 years. Sex: experimental group: 13 male (81.25%), 3 female (18.75%). Control group: 12 male (75.00%), 4 female (25.00%). Diagnosis: first episode of unilateral stroke more than 1 month prior; no progression of nervous system symptoms for more than 48 h.	Six weeks, 5 times a week.	Experimental group (MIT + conventional treatment): 16 patients. Control group (conventional treatment only): 16 patients.	FMA-LE, FIM, BBS.	Both groups showed significant improvement in FMA-LE, BBS, and FIM scores after 6 weeks. The experimental group (receiving MIT in addition to conventional therapy) showed significantly greater improvement in all three outcome measures compared to the control group.	Effect size: Significant improvement in FMA-LE, BBS, and FIM within both groups (*p* < 0.001). Significant difference in change scores between groups for FMA-LE, BBS, and FIM (*p* < 0.01). Certainty of evidence: moderate, since this is a pilot study with a small sample size, which limits the generalizability of the findings.	Guided/motor imagery intervention: Patients in the experimental group listened to a 20 min audio CD guiding them through relaxation exercises, followed by detailed imagery of simple lower limb activities (e.g., turning over, sit-to-stand, walking, stair climbing). Each activity was imagined three times. Erogation of guided imagery: The guided imagery was delivered via an audio CD, with a psychologist providing the verbal instructions. It involved imagining specific movements and activities, suggesting a combination of internal (imagining oneself performing the action) and likely both visual and kinesthetic imagery components.
Ji et al., 2021 [81]	To investigate the effects of a home-based GMI training program on upper limb motor function in patients with chronic stroke.	Study design: RCT. Size: 42 participants. Age: GMI group: mean 53.29 years. Control group: mean 61.75 years. Sex: GMI group: 9 male, 8 female. Control group: 13 male, 7 female. Diagnosis: first-ever supratentorial stroke, ≥3 months since onset.	8 weeks.	GMI group: 21 initially, 17 completed the study; control group (conventional therapy): 21 initially, 20 completed the study	MFT, FMA, MBI.	Both the GMI group and the control group showed improvements in FMA, MFT, and MBI scores over time. The GMI group showed a significantly greater improvement in upper limb MFT scores compared to the control group.	Effect size: Significant increase in FMA and MFT scores over time in both groups (*p* < 0.05). Significant difference in upper limb MFT score improvement between the GMI and control groups (*p* = 0.042). Certainty of evidence: Moderate. This is a single study with a moderate sample size and RCT design.	Guided/motor imagery intervention: The GMI intervention consisted of three components: implicit MI (left/right hand discrimination task), explicit MI (imagining movements without actually performing them), and mirror therapy (performing movements with the unaffected hand while observing the reflection in a mirror). Participants performed these tasks at home. Erogation of guided imagery: Implicit MI was conducted using visual stimuli (photographs of hands) on a smartphone. Explicit MI was also visually guided (photographs on smartphone) with instructions to imagine the movements. Mirror therapy used visual feedback from the mirror to guide movements.
Kumar et al., 2016 [82]	To evaluate the effects of combining MI with physical practice on paretic lower extremity muscle strength and gait performance in ambulant stroke subjects.	Study design: RCT. Size: 40 participants. Age: experimental group: mean 53.0 years. Control group: mean 51.0 years; Sex: experimental group: 16 male (80%), 4 female (20%); control group: 14 male (70%), 6 female (30%). Diagnosis: unilateral first episode of stroke at least 3 months prior.	3 weeks, 4 days per week.	Experimental group (physical + mental practice): 20 participants. Control group (physical practice only): 20 participants.	Isometric muscle strength of hip, knee, and ankle using a hand-held dynamometer. Self-selected 10 m gait speed.	Both groups showed significant improvement in all outcome measures after 3 weeks. The experimental group (task-oriented training plus MI) showed significantly greater improvement in paretic hip muscle strength (flexors and extensors), knee extensor strength, ankle dorsiflexor strength, and gait speed compared to the control group (task-oriented training only). These findings suggest that adding task-specific MI training to physical practice enhances muscle strength and gait performance in ambulant stroke patients.	Effect size: Significant improvement in all outcome measures within both groups (*p* < 0.05). Significant difference between groups in improvements in hip flexor strength (*p* = 0.01), hip extensor strength (*p* = 0.01), knee extensor strength (*p* = 0.01), ankle dorsiflexor strength (*p* = 0.01), and gait speed (*p* = 0.01). Certainty of evidence: Moderate. This is a single study with a moderate sample size and RCT design.	Guided/motor imagery intervention: The experimental group received 30 min of audio-based lower extremity mobility tasks for MI practice in addition to their task-oriented physical training. The audio tape included relaxation, cognitive visual images related to lower extremity task characteristics, visualization of performing the task, and experiencing kinesthetic sensations. Erogation of guided imagery: The guided imagery was delivered via audio tape, with verbal instructions and explanations of lower extremity task components. It was designed to promote both visual (imagining the movements) and kinesthetic (imagining the sensations of the movements) imagery.
Haire et al., 2021 [83]	To examine the effects of TIMP training, with and without MI, on cognitive and affective outcomes in individuals with chronic stroke.	Study design: RCT. Size: 30 participants. Age: TIMP: mean 54.7 years; TIMP + cMI: mean 55.5 years; TIMP + MI: mean 57.6 years. Sex: TIMP: 5 male, 5 female; TIMP + cMI: 5 male, 5 female; TIMP + MI: 6 male, 4 female. Diagnosis: hemiparesis following a unilateral stroke sustained > 6 months prior to enrollment; at least minimal volitional movement of the affected limb.	Three times a week for three weeks.	TIMP: 10 participants; TIMP + cMI (cued MI): 10 participants. TIMP + MI (MI): 10 participants.	TMT, DST, GSE, MAACL-R, SAM.	TIMP + MI group showed a significant improvement in mental flexibility. Both TIMP and TIMP + cMI groups showed improvements in positive affect. The TIMP + cMI group showed a decrease in negative affect. No significant improvements were found in short-term memory or self-efficacy for any group.	Effect size: Significant improvement in TMT-B for the TIMP + MI group (*p* = 0.039). Significant improvement in positive affect for TIMP and TIMP + cMI groups (*p* = 0.045 for TIMP + cMI on MAACL-R; *p* = 0.011 and *p* = 0.034 for TIMP and TIMP + cMI, respectively, on SAM). Significant decrease in negative affect for the TIMP + cMI group (*p* = 0.041). Certainty of evidence: Moderate. This is a single study with a moderate sample size and RCT design.	Guided/motor imagery intervention: Participants in the TIMP + cMI group received 30 min of TIMP followed by 15 min of cued MI (listening to a metronome beat while engaging in MI). The TIMP + MI group received 30 min of TIMP followed by 15 min of MI without external cues. Exercises were designed to train gross and fine motor control using acoustic and electronic instruments. Erogation of guided imagery: The TIMP + cMI group used auditory cues (metronome) during MI. The TIMP + MI group performed MI without external cues. In both cases, the imagery likely involved internal visual and kinesthetic components as participants imagined themselves playing the instruments.
Bovonsunthonchai et al., 2020 [84]	To investigate the effects of MI combined with SPCCT on gait and lower limb muscle strength in stroke survivors.	Study design: RCT. Size: 40 participants. Age: experimental group: mean 49.90 years. Control group: mean 55.55 years. Sex: experimental group: 15 male (75%), 5 female (25%); control group: 11 male (55%), 9 female (45%). Diagnosis: first event of stroke and unilateral involvement of the body.	4 weeks, 3 times per week.	Experimental group (MI with structured SPCCT): 20 participants. Control group (health education with SPCCT): 20 participants.	Temporo-spatial gait variables (step length, step time, stride length, gait speed, cadence) using a 2D motion analysis system. Muscle strength of the affected hip flexor and extensor, knee flexor and extensor, ankle dorsiflexor and plantarflexor using a hand-held dynamometer.	Both groups showed significant improvements in gait and muscle strength over time. The experimental group (MI + SPCCT) showed significantly greater improvements in several gait variables (step length, stride length, gait speed, cadence) and some muscle strength measures (hip flexor, knee extensor) compared to the control group (health education + SPCCT).	Effect size: Significant effect of time (*p* < 0.001) for most gait variables in both groups. Significant interaction effect of group by time (*p* < 0.05) for several gait variables (step length, stride length, gait speed, cadence) and some muscle strength measures (hip flexor, hip extensor, knee flexor). Significant effects even at 4 weeks post interventions. Certainty of evidence: Moderate. This is a single study with a moderate sample size and RCT design.	Guided/motor imagery intervention: The experimental group received 25 min of MI training (visual and kinesthetic imagery of lower extremity movements) followed by 65 min of SPCCT. The MI training progressed in complexity (speed, rhythm) and was monitored using a heart rate monitor and observation of finger movement Erogation of guided imagery: The MI training involved both visual (imagining the movements) and kinesthetic (imagining the sensations) imagery, performed in a first-person perspective with eyes closed. The training was delivered verbally by the researcher, guiding participants through the imagery process.
Aung et al., 2022 [85]	To examine the effects of MI combined with SPCCT on dynamic balance, endurance, and functional mobility in post-stroke individuals.	Study design: RCT. Size: 40 participants. Age: experimental group: mean 49.40 years; control group: mean 55.55 years. Sex: experimental group: 15 male (75%), 5 female (25%); control group: 11 male (55%), 9 female (45%). Diagnosis: first stroke with unilateral hemiparesis.	4 weeks, 3 sessions per week.	Experimental group (task-oriented MI and circuit class training): 20 participants; control group (health education and circuit class training): 20 participants	Step test (affected and unaffected limb); 6 MWT and TUG.	Both groups showed significant improvements in all functional mobility outcomes over time. The experimental group (MI + SPCCT) showed significantly greater improvements in the step test (affected limb), 6 MWT, and TUG compared to the control group (health education + SPCCT).	Effect size: Significant main effect of time (*p* < 0.001) for all functional mobility outcomes. Significant interaction effect of group by time (*p* < 0.001 for step test affected limb, 6 MWT; *p* = 0.009 for TUG). Significant differences between groups at T2 (4 weeks) for step test (affected and unaffected limb) and TUG (*p* < 0.05). Certainty of evidence: Moderate. This is a single study with a moderate sample size and RCT design.	Guided/motor imagery intervention: The experimental group received 25 min of MI (kinesthetic and visual imagery) followed by 65 min of SPCCT. The MI was performed in three stages: relaxation, imagery practice (kinesthetic and visual), and refocusing. A metronome was used for progression in the stepping task. Erogation of guided imagery: The MI involved both kinesthetic (imagining the sensation of movement) and visual (imagining seeing oneself perform the movement) imagery, delivered from a first-person perspective. Participants closed their eyes during the practice. The imagery was guided by a script and the engagement was monitored through finger counting and pulse rate monitoring.
Wang et al., 2023 [86]	To explore the unique MIT-related brain reorganization in stroke patients using task-based fMRI.	Study design: RCT. Size: 39 participants. Age: 18–80 years. Sex: 36 males and 3 females. Diagnosis: first-ever stroke (infarct or hemorrhage); stroke onset between 3 and 12 months before enrollment.	4 weeks, 5 days a week.	MIT group: 22 participants. Control group: 17 participants.	FM-UL, BI, and fMRI.	Both groups showed improvement in FM-UL, but the MIT group showed significantly greater improvement compared to the control group. Only the MIT group showed significant improvement in BI. fMRI analysis revealed that MIT helped decrease compensatory activation in both hemispheres and reshape FC within the ipsilesional hemisphere.	Effect size: Significant group × time interaction for FM-UL (F1,36 = 35.27, *p* < 0.001) and BI (F1,36 = 8.79, *p* = 0.007). FMA-UL improvement was significantly greater in the MIT group (*p* < 0.001). Significant group × time interaction for activation in contralesional S1 (*p* = 0.031) and ipsilesional M1 (*p* = 0.043). Significant group × time interaction for FC between ipsilesional M1 and IPL (*p* < 0.001) and ipsilesional M1 and putamen (*p* = 0.004). Certainty of evidence: Moderate. This is a single study with a moderate sample size. The randomized, controlled design strengthens the study, but the single-blind nature (therapists not blinded) and specific inclusion/exclusion criteria might limit generalizability.	Guided/motor imagery intervention: The MIT group received 30 min of MIT daily, consisting of relaxation (3 min), imagery of basic movements (10 min), imagery of progressive task-oriented training/ADLs (15 min), and refocusing (3 min). First-person MIT was used, with standardized verbal instructions and individualized tasks. The therapist checked patient engagement in the imagery. Erogation of guided imagery: The MIT involved both kinesthetic and visual imagery, delivered from a first-person perspective. The therapist provided standardized verbal instructions to guide the imagery.
Wang et al., 2020 [87]	To investigate the whole-brain reorganization associated with MIT in stroke patients using resting-state fALFF.	Study design: RCT. Size: 31 participants. Age: MIT group: mean age 53.38 years; control group: mean age 60.47 years. Sex: MIT group: 16 male, 0 female (initially 16 male, 1 female). Control group: 14 male, 1 female (initially 16 male, 1 female). Diagnosis: stroke.	4 weeks, 5 days per week.	MIT group: 16 participants; control group: 15 participants.	FM-UL, MBI. Resting-state fMRI: fALFF and FC.	While both groups improved in MBI, the difference between groups was not statistically significant. fMRI analysis revealed increased fALFF in the ipsilesional IPL in the MIT group, which was positively correlated with FM-UL improvement. Changes in FC between the ipsilesional IPL and other brain regions were also observed and related to motor recovery.	Effect size: Significant group × time interaction for FM-UL (F(1, 29) = 53.75, *p* < 0.001) and MBI (F(1, 29) = 16.16, *p* < 0.001). Significant time × group interactions for fALFF in contralesional inferior frontal gyrus, inferior temporal gyrus, and ipsilesional IPL and middle temporal gyrus. Significant time × group interactions for FC between the ipsilesional IPL and several other brain regions. Significant positive correlation between fALFF change in the ipsilesional IPL and FM-UL improvement (ρ = 0.67, *p* < 0.001). Certainty of evidence: Moderate. This is a single study with a moderate sample size and RCT design.	Guided/motor imagery intervention: Patients in the MIT group received 30 min of specific MIT daily after 3 h of conventional rehabilitation therapy. Erogation of guided imagery: it was delivered by blinded therapists.
Choi et al., 2022 [88]	To investigate the effect of AO combined with MI intervention on upper extremity function and corticospinal activation in stroke patients.	Study design: RCT. Size: 45 participants. Age: experimental group: mean age 62.68 years; control group: mean age 63.43 years. Sex: experimental group: 12 male, 10 female; control group: 12 male, 11 female. Diagnosis: subacute patients with a stroke onset period of 2 to 8 months.	8 weeks, 5 times a week, 25 min per session.	Experimental group (AO with MI): 22 participants. Control group (AO only): 23 participants.	MEP, FMA-UE, WMFT, MAL.	Both groups showed significant improvement in all upper extremity function measures. The experimental group (AO + MI) showed significantly greater improvement in FMA UE and MAL AOU compared to the control group (AO only). While both groups showed significant changes in MEP amplitude, the improvement was significantly greater in the experimental group.	Effect size: Significant group × time interaction for FMA UE (*p* = 0.002) and MAL AOU (*p* = 0.022). Significant difference in the amount of change before and after intervention for FMA UE (*p* = 0.000) and MAL AOU (*p* = 0.000) in the experimental group compared to the control group. Significant difference in the amount of change before and after intervention for MEP amplitude (*p* = 0.001) in the experimental group compared to the control group. Certainty of evidence: Moderate. This is a single study with a moderate sample size and RCT design.	Guided/motor imagery intervention: The experimental group performed AO in parallel with MI. Participants selected five purposeful daily life activities they wanted to perform from a list of ten. They watched videos of these activities while simultaneously imagining themselves performing the movements. Each task was performed for 4 min with a 1 min rest period, totaling 25 min of intervention. The therapist instructed them to imagine how their upper extremities and hands would move, concentrating on the task. Erogation of guided imagery: Participants imagined themselves performing the selected tasks while watching the videos (AO). The imagery was likely both visual (seeing the actions) and kinesthetic (imagining the feeling of the movements). The videos were presented from a first-person perspective.
Li et al., 2014 [89]	To investigate the role of an MI-BCI training combined with FES for stroke patient rehabilitation, focusing on classification accuracy, rehabilitation outcomes, and neurophysiological changes.	Study design: RCT. Size: 15 participants. Age: 50–80 years. Sex: 11 males and 4 females. Diagnosis: stroke occurrence 1–6 months prior to the study.	8 weeks, 3 times per week, 1–1.5 h per session (BCI group), 20 min of FES 3 times per week (control group), conventional therapies 5 times per week for both groups.	BCI group: 8 participants. Control group: 7 participants.	FMA, ARAT, EEG, and ERD.	The BCI group showed significant improvement in online CA after training. Both groups showed significant improvement in FMA and ARAT scores, but the BCI group showed greater improvement in ARAT after 6 weeks. The BCI group demonstrated stronger ERD in the affected SMC after training.	Effect size: Significant increase in CA in the BCI group (*p* = 0.002) and higher CA in the BCI group compared to the control group post-training (*p* = 0.018). Significant increase in FMA and ARAT scores in both groups (*p* = 0.00 for both). Significant difference in ARAT scores between groups after 6 weeks (*p* < 0.05). Significant difference in ERD of the affected SMC in the BCI group post-training (*p* < 0.05). Certainty of evidence: Moderate. This is a single study with a small sample size and RCT design.	Guided/motor imagery intervention: The BCI group underwent MI training before the BCI-FES intervention. Patients were instructed on MI skills, practiced imagining drinking water (the assigned MI task), and were introduced to the task via video, performing it first with the unaffected limb. Kinesthetic imagery was emphasized. During the BCI-FES training, patients played games that required them to imagine movements of the affected or unaffected limb. Correct MI (based on >60% online CA) triggered FES to the affected limb’s extensor carpus radialis muscles, along with visual and auditory feedback. Erogation of guided imagery: Patients were instructed to perform kinesthetic imagery of specific movements, primarily imagining drinking water. The MI tasks within the BCI-FES intervention involved imagining movements needed for the games, which were visually presented. It is implied that the imagery was first-person, as patients were imagining their own limb movements.
Butler et al., 2006 [90]	To explore the feasibility of using mental practice in conjunction with CIMT to improve upper extremity performance in stroke patients and to examine the associated cortical changes.	Study design: RCT. Size: 4 participants. Age: 51–73 years. Sex: 3 men and 1 woman. Diagnosis: 3 months post-stroke.	2 weeks.	Mental practice and physical therapy (mental practice + CIMT): 2 patients; mental practice only: 1 patient; physical therapy (CIMT) only: 1 patient.	WMFT, MAL, MIQ-R, VMIQ. Sirigu break test, fMRI to assess cortical activations.	CIMT only: significant WMFT and MAL improvement; increased bilateral motor/premotor activation. Mental practice only: minor functional gains; contralateral motor activation (executed task) and bilateral inferior/middle temporal activation (imagery). CIMT + mental practice: functional and imagery improvements; focal contralateral M1 activation (execution) and ipsilateral occipital/temporal activation (imagery).	Effect size: not provided. Certainty of evidence: Low. This is a very small pilot study with limited generalizability due to the small sample size and heterogeneity of the participants.	Guided/motor imagery intervention: the mental practice intervention consisted of 3 h per day for 2 consecutive weeks, similar in duration to the CIMT intervention. Erogation of guided imagery: not specified.
Wang et al., 2019 [91]	To investigate the brain reorganization after MIT and CRT in patients with subcortical stroke, focusing on FC patterns and graph-theory parameters.	Study design: RCT. Size: 31 participants. Age: 18–80 years. Sex: MIT group: 16 male, 1 female (initially 17 total); CRT group: 16 male, 1 female (initially 17 total). Diagnosis: first-ever subcortical ischemic or hemorrhagic stroke between 3 and 12 months post-stroke.	4 weeks, 5 days a week, 3 h of CRT and 30 min of MIT (or health education for the control group) per day.	MIT group: 17 participants (initially recruited, but one excluded due to head motion)—final sample: 16; CRT group: 17 participants (initially recruited, two excluded due to head motion)—final sample: 15.	FM-UL and resting-state fMRI: FC and graph-theory parameters (clustering coefficient and characteristic path length).	The MIT group showed greater improvement in FM-UL scores compared to the CRT group. Both groups showed increased inter-hemispheric FC and decreased intra-hemispheric FC of the ipsilesional M1, but the MIT group showed a specific increase in FC within ipsilesional precentral and postcentral gyri, middle cingulate gyrus, and supramarginal gyrus. The clustering coefficient increased significantly in the MIT group, and this increase was positively correlated with FM-UL improvement.	Effect size: Significant difference in FM-UL improvement between groups (*p* < 0.001). Significant group × time interaction for FC in ipsilesional precentral and postcentral gyri, middle cingulate gyrus, and supramarginal gyrus. Significant group × time interaction for the clustering coefficient (*p* = 0.02). Significant positive correlation between the increase in the clustering coefficient and FM-UL improvement (R = 0.42, *p* = 0.02). Certainty of evidence: Moderate. This is a single study with a moderate sample size and RCT design.	Guided/motor imagery intervention: Patients in the MIT group received 30 min of supervised MIT after 3 h of standard CRT. The MIT protocol consisted of relaxation, imagining basic movements, imagining goal-directed movements/ADLs, and refocusing. First-person MIT of the affected upper extremity was used. The therapist guided the training and checked patient engagement. Erogation of guided imagery: The MIT involved first-person imagery of movements of the affected upper extremity. A combination of visual (imagining seeing the movement) and kinesthetic (imagining the feeling of the movement) imagery, guided by verbal instructions from the therapist.
Sun et al., 2016 [92]	To examine the effects of MIT combined with CRT on motor function and brain activation in chronic stroke patients with severe hemiparesis, and to explore the neural reorganization mechanisms underlying motor recovery.	Study design: RCT. Size: 18 participants. Age: MIT group: 56.67 ± 12.67 years; CRT group: 56.11 ± 10.83 years. Sex: MIT group: 8 male, 1 female; CRT group: 9 male, 0 female. Diagnosis: only one stroke (infarct or hemorrhage); stroke experienced 3–6 months prior to study enrollment.	4 weeks, 5 days a week, 3 h of CRT and 30 min of MIT (or usual activities for the control group) per day.	MIT group: 10 participants initially; 9 completed the study. CRT group: 10 participants initially; 9 completed the study.	FM-UL and fMRI during a passive finger flexion-extension task.	The MIT group showed significantly greater improvement in FM-UL scores compared to the CRT group. Two patterns of cortical reorganization were observed: increased activation in the cSMC in most patients and focusing of activation in the cSMC with increased laterality index in a smaller portion of patients. A positive correlation was found between increases in cSMC activation and FM-UL score improvements.	Effect size: Significant difference in FM-UL score changes between groups (*p* = 0.004). Significant positive correlation between relative ΔcSMC and relative ΔFM-UL (R = 0.68, *p* = 0.02). Certainty of evidence: Low. This is a single study with a relatively small sample size after dropouts.	Guided/motor imagery intervention: Patients in the MIT group received 30 min of MIT daily, consisting of relaxation, imagining simple movements, imagining complex ADLs, and refocusing. Kinesthetic, first-person imagery was emphasized. Erogation of guided imagery: the therapist monitored the training and checked patient engagement.
Hong et al., 2012 [93]	To compare the effects of MIT-EMG and FES on motor function and cerebral glucose metabolism in chronic stroke patients.	Study design: RCT. Size: 14 participants. Age: mean age 51.29. Sex: 9 males and 5 females. Diagnosis: stroke.	4 weeks, 5 days a week, two 20 min sessions per day.	MIT combined with EMG-triggered electrical stimulation (MIT-EMG) group: 7 participants; FES group: 7 participants.	FMA-UE, MAL, MBI, and PET scans to measure cerebral glucose metabolism.	MIT-EMG showed greater improvement in the FMA-UE scores compared to FES. The MAS improved in the MIT-EMG group but not the FES group. PET scans showed increased cerebral glucose metabolism in the supplementary motor, precentral, and postcentral gyri of the contralesional hemisphere after MIT-EMG, but not after FES.	Effect size: Significant difference in FMA-UE score changes between groups (*p* = 0.01). Significant improvement in FMA and MAS within the MIT-EMG group (*p* < 0.05). Certainty of evidence: Moderate. This is a small study with 14 participants. While it is an RCT, the small sample size limits the generalizability of the findings.	Guided/motor imagery intervention: The mental imagery used in MIT-EMG was a simple movement (vigorous waving of the entire arm). The intervention consisted of three stages: mental imagery (maximum 12 s), stimulation (6 s), and relaxation (12 s), guided by the instrument’s monitor. Erogation of guided imagery: participants were instructed by a therapist to imagine the feeling and movement of their arm.
Hong et al., 2017 [94]	To investigate the effects of combining tDCS with MI-BCI training on motor function, white matter integrity, and CBF in chronic stroke patients.	Study design: RCT. Size: 19 participants. Age: tDCS group: 52.8 ± 12.3 years; control group (sham-tDCS group): 56.4 ± 9.6 years. Sex: tDCS group: 4 female, 5 male; sham-tDCS group: 1 female, 8 male. Diagnosis: first-ever subcortical stroke more than 9 months prior to study enrollment, leading to unilateral moderate to severe upper extremity impairment.	Two weeks, ten 40 min MI-BCI training sessions, each preceded by 20 min of tDCS or sham-tDCS.	tDCS group: 9 participants; control group (sham-tDCS group): 9 participants.	FMA. Resting motor threshold measured by single-pulse TMS. DTS to assess white matter integrity. Pseudo-continuous arterial spin labeling MRI to measure CBF.	Both MI-BCI training with tDCS and MI-BCI training with sham-tDCS improved motor function (FMA scores), but there was no significant difference between the groups. tDCS combined with MI-BCI led to increased fractional anisotropy in the ipsilesional corticospinal tract and corpus callosum, mainly due to decreased radial diffusivity. tDCS led to a long-lasting decrease in CBF and RMT in the ipsilesional sensorimotor cortex and altered CBF patterns compared to sham stimulation.	Effect size: Significant time effect on FMA scores (*p* < 0.0001). Significant increase in FA in the tDCS group compared to the sham group (*p* < 0.05). Significant difference in CBF changes between groups in the ipsilesional somatosensory cortex (*p* = 0.0186). Certainty of evidence: Moderate. This study has several limitations, including the relatively small sample size, particularly for the CBF analysis, and the fact that MEPs were not detectable in a subset of participants.	Guided/motor imagery intervention: The MI-BCI training involved mental imagery of a reaching task. Motor intention was detected using EEG, which triggered movement of the affected arm using a robot. Erogation of guided imagery: The mental imagery task involved imagining a reaching movement. The specific type of imagery (visual, kinesthetic, etc.) is not explicitly stated, but it is likely that participants were instructed to imagine the movement and the feeling of reaching.
Li et al., 2018 [95]	To investigate the effect of MI training combined with traditional rehabilitation treatment on hand function, MEP, and white matter integrity in stroke patients.	Study design: RCT. Size: 20 participants. Age: 18–80 years. Sex: MP group: 8 male, 2 female; PP group: 7 male, 3 female. Diagnosis: stroke.	4 weeks, 5 days a week, 45 min of MIT or PP group after traditional rehabilitation training.	MP group: 10 participants. PP group: 10 participants.	Action Research Arm Test, FMA-UE, MEPs amplitude and latency in the abductor pollicis brevis muscle, FA and mean diffusivity of white matter pathways measured by DTI.	Both groups showed improvement in hand function, but the MP group showed significantly greater improvement than the PP group. MEP amplitude improved significantly in the MP group compared to the PP group after training. DTI showed significant increases in FA in the dorsal pathways in the MP group, suggesting improved white matter integrity.	Effect size: Significant difference in Action Research Arm Test scores between groups post-training (*p* < 0.00). Significant difference in FMA scores between groups post-training (*p* = 0.001). Significant difference in MEP amplitude between groups post-training (*p* = 0.0181). Certainty of evidence: Moderate. This is a pilot study with a relatively small sample size. While the RCT is a strength, the specific inclusion/exclusion criteria and the lack of detailed reporting on some demographic data may limit the generalizability of the findings.	Guided/motor imagery intervention: Patients imagined 11 small hand tasks (e.g., thumb flexion–extension, making a fist) in a first-person perspective. They were guided by watching videos, therapist demonstrations, and performing the movements with their unaffected hand before imagining the movement with the affected hand. Erogation of guided imagery: The MI was performed in a first-person perspective. Patients were instructed to imagine the feeling of the muscle and skin moving during the imagined tasks. Visual input was provided through videos and demonstrations, and kinesthetic input was provided by performing the movements with the unaffected hand.

Legend: motor imagery (MI), brain–computer interface (BCI), randomized controlled trial (RCT), Fugl–Meyer assessment (FMA), Fugl–Meyer Assessment of the Upper Extremity (FMA-UE), functional magnetic resonance imaging (fMRI), Z-normalized Amplitude of Low-Frequency Fluctuations (zALFF), Z-normalized Regional Homogeneity (zReHo), National Institutes of Health Stroke Scale (NIHSS), Medical Research Council (MRC), Modified Ashworth Scale (MAS), electroencephalography (EEG), Berg Balance Scale (BBS), Functional Reach Test (FRT), timed up-and-go test (TUGT), Movement Imagery Questionnaire (MIQ-3), Fugl–Meyer Assessment (FMMA), transcranial direct current stimulation (tDCS), event-related desynchronization (ERD), near-infrared spectroscopy (NIRS), Action Research Arm Test (ARAT), Motor Activity Log (MAL), Kinesthetic and Visual Imagery Questionnaire (KVIQ-10), Attention Network Test (ANT), Wolf Motor Function Test (WMFT), Modified Barthel Index (MBI), Symbol Digit Modalities Test (SDMT), functional electrical stimulation (FES), Fugl–Meyer motor assessment UL (FMA-UL), motor-evoked potentials (MEPs), visual–motor imagery (VMI), functional near-infrared spectroscopy-mediated neurofeedback (fNIRS-NFB), Functional Independence Measure (FIM), Fugl–Meyer motor assessment (F-M), supplementary motor area (SMA), electrical stimulation (ES), low-frequency repetitive transcranial magnetic stimulation (LF-rTMS), finger tapping test (FFT), Medical Research Council (MRC), Box and Block Test (BBT), Stroke Impact Scale (SIS), MRC scale score for wrist extensor (MRC-WE), active range of motion in wrist extension (AROM-WE), Fugl–Meyer Assessment of Lower Extremity (FMA-LE), Functional Ambulation Category Scale (FAC), virtual reality (VR), repetitive transcranial magnetic stimulation (rTMS), activities of daily living (ADL), motor imagery training (MIT), muscle relaxation (MR), Movement Imagery Questionnaire—Revised (MIQ-RS), autonomic nervous system (ANS), action observation (AO), action execution (AE), heart rate variability (HRV), natural logarithm of the root mean square of successive differences of heartbeats (lnRMSSD), Hospital Anxiety and Depression Scale (HADS), Mental Status Questionnaire (MSQ), integrated motor imagery (IMI), Falls Efficacy Scale Swedish version (FESS), step activity monitor (SAM), task-oriented circuit class training (TOCCT), Functional Ambulation Classification (FAC), Rivermead Visual Gait Assessment (RVGA), 6-minute walk test (6 MWT), Walking Ability Questionnaire (WAQ), graded motor imagery (GMI), Manual Function Test (MFT), therapeutic instrumental music performance (TIMP), Trail Making Test (TMT), Forward Digit Span Test (DST), General Self-Efficacy Scale (GSE), Multiple Affect Adjective Check List—Revised (MAACL-R), Self-Assessment Manikin (SAM), structured progressive circuit class therapy (SPCCT), Fugl–Meyer Assessment Upper Limb subscale (FM-UL), functional connectivity (FC), Primary Somatosensory Cortex (S1), primary motor cortex (M1), inferior parietal lobule (IPL), fractional Amplitude of Low-Frequency Fluctuations (fALFF), magnetic resonance imaging (MRI), Amount of Use (AOU), sensorimotor cortex (SMC), classification accuracy (CA), constraint-induced movement therapy (CIMT), Vividness of Movement Imagery Questionnaire (VMIQ), conventional rehabilitation therapy (CRT), contralateral sensorimotor cortex (cSMC), electromyography-triggered electrical stimulation (MIT-EMG), Positron Emission Tomography (PET), cerebral blood flow (CBF), diffusion tensor imaging (DTI), transcranial magnetic stimulation (TMS), usual activities (PP group), fractional anisotropy (FA); motor imagery training combined with traditional rehabilitation treatment (MP).

## Data Availability

Not applicable.
